# Study on the fracture characteristics controlled by filling ratio in cut-and-fill mining

**DOI:** 10.1371/journal.pone.0354444

**Published:** 2026-10-16

**Authors:** Qingju Qin, Zhiqiang Wang, Changxiang Wang, Jiahao Wang, Baoliang Zhang

**Affiliations:** 1 School of Mines, China University of Mining and Technology, Xuzhou, China; 2 Qianyingzi Coal Mine, Anhui Hengyuan Coal Industry and Electricity Power Co., Ltd., Wanbei Coal-Electricity Group, Suzhou, China; 3 State Key Laboratory for Safe Mining of Deep Coal Resources and Environment Protection, Anhui University of Science and Technology, Huainan, China; 4 School of Safety Science and Engineering, Anhui University of Science and Technology, Huainan, China; 5 Jinfeng Coal Mine, National Energy Group Ningxia Coal Industry Co., Ltd., Yanchi, China; 6 School of Architecture & Civil Engineering, Liaocheng University, Liaocheng, China; The University of Hong Kong, HONG KONG

## Abstract

To mitigate surface subsidence and stope instability induced by an insufficient filling ratio in cut‑and‑fill mining, this study investigates overlying strata fracture characteristics and instability mechanisms by means of similar material simulation, PFC numerical modeling, and theoretical analysis. Filling ratio acts as the dominant factor for overburden and stope stability: full (100%) filling suppresses roof displacement and key-stratum breakage to realize reliable gangue-pillar bearing and overburden control, whereas 50% insufficient filling triggers key-stratum fracture and domino-style chain failure starting from central goaf gangue pillars, further producing severe overburden destruction and large-magnitude surface subsidence. PFC simulations prove cut-and-fill mining forms a composite three-arch structure, whose evolution under gangue compaction controls overburden damage and surface subsidence. Analysis confirms the criticality of middle gangue pillar stability, so Cut-and-fill mining design should strengthen middle and end gangue pillars. This study clarifies filling ratio control mechanisms, providing technical support for filling parameter optimization and safe, efficient Cut-and-fill mining.

## 1. Introduction

For a long time, the caving method has been the dominant coal mining approach. Despite its certain production efficiency, it causes severe surface subsidence, which damages the land and groundwater environment and threatens the ecosystem significantly [1–3]. In recent years, green mining has been recognized as the primary solution to mitigate these issues, among which gangue filling—which realizes backfilling of coal gangue within goafs—is a key green mining technology [4–7]. After nearly 20 years of development, gangue filling technology has gone through four generations, with continuous upgrading of hydraulic supports for solid filling coal mining. Currently, an underground integrated production system can be constructed, integrating coal mining, coal-gangue separation, gangue filling, and functions like strata movement active control, gob-side entry retaining, gas extraction, disaster prevention, and water-preserved mining. This forms the coal‑oriented collaborative production mode of “Mining‑Selection‑Filling + X” (control, retention, extraction, prevention, protection) [8–14]. Presently, this mode has achieved parallel mining-filling operations and integrated mining-filling equipment, while facing main challenges of insufficient filling materials and limited filling scale [15–17].

Considering the challenge of material scarcity in gangue filling operations, a novel approach involving Cut-and-fill mining within goaf areas is proposed, emphasizing surface subsidence control [18–20]. Research demonstrates that, alongside the mechanical properties of the filling body, its geometric dimensions constitute a critical determinant of filling efficacy. Prevailing studies predominantly concentrate on filling rates or compaction rates along the vertical axis. Employing the concept of equivalent mining height, Huang et al. [21,22] postulated that a compaction rate of 15% for the filling body is requisite to maintain surface subsidence within permissible limits. Zhou et al. [23] identified three pivotal parameters influencing the filling rate: the magnitude of pre-subsidence roof displacement, the extent of roof under-contact, and the degree of compression deformation within the filling body. Zhang et al. [14,24] established the theoretical foundation for designing the solid filling rate and analyzed its principal controlling factors. Utilizing FLAC3D simulation, Zhou et al. [25,26] revealed the stress distribution and energy characteristics within a hard roof under diverse roof control filling rates. Li et al. [27,28] simulated the grouting filling process in caving zones via PFC2D, concluding that a critical filling rate of 15% exists; below this threshold, effectively controlling roof subsidence and consequent surface subsidence proved challenging. Zhang et al. [29] investigated the collaborative management of filling rates in ultra-close coal seams through integrated physical simulation, numerical simulation, and the probability integral method. Results indicate that achieving an 80% filling rate concurrently in both the upper and lower coal seams ensures the stability of surface structures during coal pillar recovery.

As a classic strata-control theory for underground mining, stress-arch theory can well interpret load redistribution and transfer behaviours within fractured overburden. Accordingly, this paper introduces the composite stress-arch theoretical framework to characterise heterogeneous load transfer among discrete gangue pillars under Cut‑and‑fill mining conditions, which provides a theoretical premise for establishing the subsequent overburden structural evolution model.

Insufficient filling ratio will destroy the mechanical balance of the overlying rock in the stope and induce progressive failure [28,29]. However, existing studies mainly focus on the vertical filling rate or compaction rate, and there is a lack of systematic research on the influence of filling ratio on the deformation and failure of gangue pillars and the spatial-temporal evolution law of overlying rock fracture. In particular, the intrinsic mechanism of chain instability of gangue pillars caused by insufficient filling ratio and the evolution characteristics of the stress arch structure in the stope have not been fully clarified. Aiming at these research gaps, this study takes the 2−2 upper coal seam in Ordos City as the engineering background, and combines similar material simulation, PFC numerical simulation and theoretical analysis to carry out in-depth research on the fracture characteristics controlled by filling ratio in Cut-and-fill mining. The specific research contents include: exploring the deformation and failure laws of gangue pillars and overlying rock under different filling ratios, establishing the structural model of gangue pillar-roof system, summarizing the types of overlying rock failure spatial structures and the evolution law of ‘composite stress arch’. The research results are expected to enrich the theoretical system of Cut-and-fill mining and provide important theoretical basis and engineering reference for the optimization of filling parameters, prevention of dynamic disasters and control of surface subsidence in Cut-and-fill mining.

This work makes three main incremental contributions to fill the above-mentioned research gaps. Most previous studies mainly concentrate on vertical compaction-based filling rate, while this paper focuses on horizontal filling ratio. First, we quantitatively compare overburden fracture and progressive failure behaviours under 100% and 50% filling‑ratio conditions, and systematically reveal the spatiotemporal overburden fracture evolution as well as the domino-style chain-instability mechanism of gangue pillars, and reveal the domino-style chain-instability mechanism initiated by the central goaf gangue pillar under insufficient filling ratio. Second, mesoscopic PFC simulation clarifies force-chain evolution differences between full-filling and under-filling scenarios, identifying the essential role of central gangue-pillar stability for overall stope safety. Third, this study proposes a composite three-arch stress-structure evolutionary model for Cut-and-fill mining, which explains how gangue-pillar compaction drives overburden damage and surface subsidence. These findings can support parameter optimisation and pillar reinforcement design for field engineering applications.

## 2. Similar simulation

### 2.1. Engineering background

The study area is in Tongchuan Town, Dongsheng District, Ordos City, Inner Mongolia. The target coal seam has a strike length of 1629.5 m, dip length of 4132.3 m, average thickness ~4 m, and average burial depth ~100 m. Its dip angle ranges 1° ~ 5°, averaging 3°, with a simple structure, no water inrush or gas risks, and stable mining conditions.

The working face uses comprehensive mechanized full-height mining via inclined longwall retreating, with roof managed by full caving. Geological exploration shows roof and floor lithology as sandstone and sandy mudstone. Physical and mechanical parameters from coring data were compiled and analyzed. Mine formation details are in Table 1.

**Table 1:** Formation parameters of the study area.

Numbering	Lithology	Thickness /m	Body force /kN/m^3^	Elastic modulus /GPa	Compressive strength /MPa	Tensile strength /MPa	Angle of internal friction/°	Force of cohesion /MPa
R8	Conglomerate	44	22	1.30	11.90	1.13	31	1.11
R7	Sandy mudstone	12	22	1.67	16.23	1.31	28	1.24
R6	Medium grained sandstone	24	24	2.83	21.23	1.90	33	1.93
R5	Sandy mudstone	5	20	1.67	16.23	1.33	28	1.21
R4	Sandstone	8	26	3.35	28.43	2.10	33	2.14
R3	Sandstone	9	26	3.35	28.43	2.14	33	2.12
R2	Sandy mudstone	5	22	1.67	16.23	1.61	28	1.23
R1	Sandy mudstone	9	22	1.67	16.23	1.63	28	1.24
C	2−2 upper coal	4	14	1.44	10.21	1.21	30	1.02
F	Sandstone	14	26	3.35	28.43	2.14	33	2.12

It lists thickness, body force, elastic modulus, strength and other mechanical parameters of each stratum from overlying conglomerate to floor sandstone, providing basic data for similar material simulation.

The coal mine working face layout and comprehensive stratum histogram of the mining area are shown in Fig 1.

**Fig 1:**
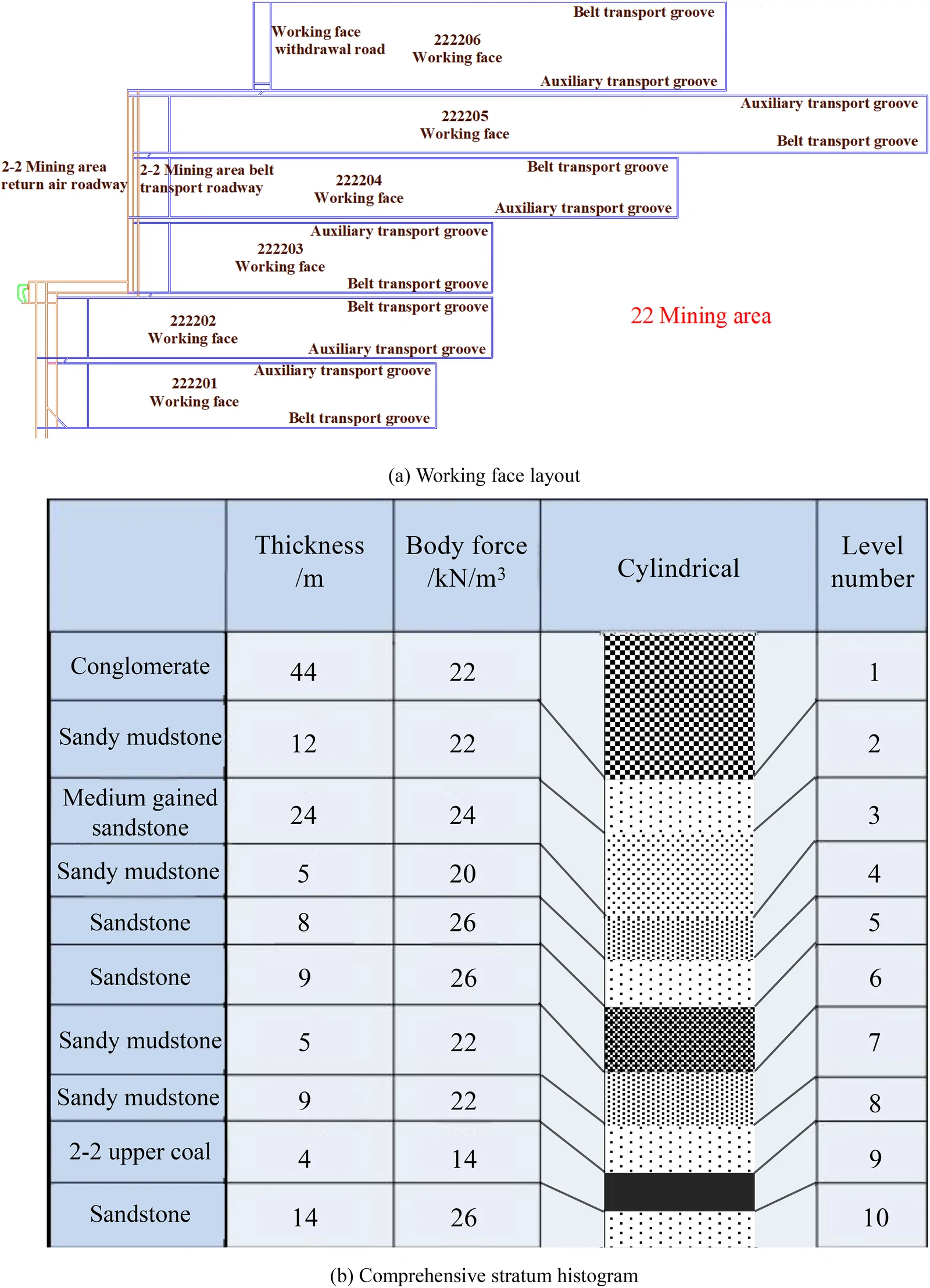
Working face layout and comprehensive strata histogram. (a) Planar layout of working faces in the 22 mining area; (b) comprehensive stratigraphic column showing the lithology, thickness, unit weight, and level number of each stratum.

The 22 mining area is mined via the 2−2 upper coal seam roadway. The working face groove is vertically arranged with the roadway, and the cut hole is parallel to it. Six fully mechanized mining faces are arranged in the area, with 25 m coal pillars left between two faces. Each face has two grooves: Belt transport and Auxiliary transport, both driven along the coal seam floor. The Belt transport groove crosses the 2−2 upper coal seam auxiliary, connects with the return air roadway, and links to the Belt transport roadway through the coal chute, mainly for coal transportation and return air. The auxiliary transport groove directly connects with the auxiliary transport roadway, mainly for material transportation and air intake.

To identify the key bearing stratum of the overlying rock layers, the interlayer load of each rock layer in the study area was calculated and analyzed. The results show that the R6 medium grained sandstone is the main key stratum controlling the movement of the entire overlying rock layers and the load transfer [30–32].

The classical key‑stratum discrimination method is adopted in this work. The inter‑layer load exerted by the overlying strata on the underlying rock stratum is calculated by:


$$\begin{array}{c} q_{i}(x)=\frac{E_{i}h_{i}^{\text{3}}\sum_{k=i}^{n}\gamma_{k}h_{k}}{\sum_{k=i}^{n}E_{k}h_{k}^{\text{3}}} \end{array}$$

Where $q_{i}$ is the inter‑layer load acting on stratum i; $E_{i}$ is elastic modulus of stratum i; $h_{i}$ is stratum thickness; $\gamma_{k}$ is unit weight of stratum k.

Two core criteria for judging main key stratum are followed: (1) Stiffness condition: when $q_{i}<q_{i+\text{1}}$, stratum i satisfies the stiffness‑key‑layer condition; (2) Strength condition: the breaking interval of this stratum is larger than the breaking interval of all overlying subordinate key strata. According to the measured lithological parameters in Table 1, iterative inter‑layer‑load calculation indicates that R6 medium‑grained sandstone satisfies both stiffness and strength criteria. Its fracture span is larger than that of upper rock strata, and it bears the load of most overlying strata. Therefore, R6 medium‑grained sandstone is identified as the main key stratum.

### 2.2. Experimental design

In this similar material simulation experiment, similar materials (sand, calcium carbonate, gypsum, water, etc.) were used to make a physical model similar to the field engineering conditions in accordance with the similar material theory and similarity criteria. The similarity ratio of dimensions is 1:100, the similarity ratio of unit weight is 1:1.5, and the similarity ratio of strength is 1:150. As shown in Fig 2, the geometric dimensions of the model are 190 cm in length, 22 cm in width, and 134 cm in laying height. The corresponding similar material ratio scheme was selected according to the compressive strength parameters of each stratum [29,33]. Latitude and longitude grids with a size of 10 cm × 10 cm were drawn on the front of the model with ink lines, and drawing pins were inserted at the grid intersections as displacement observation points. A total station was used for displacement monitoring.

**Fig 2:**
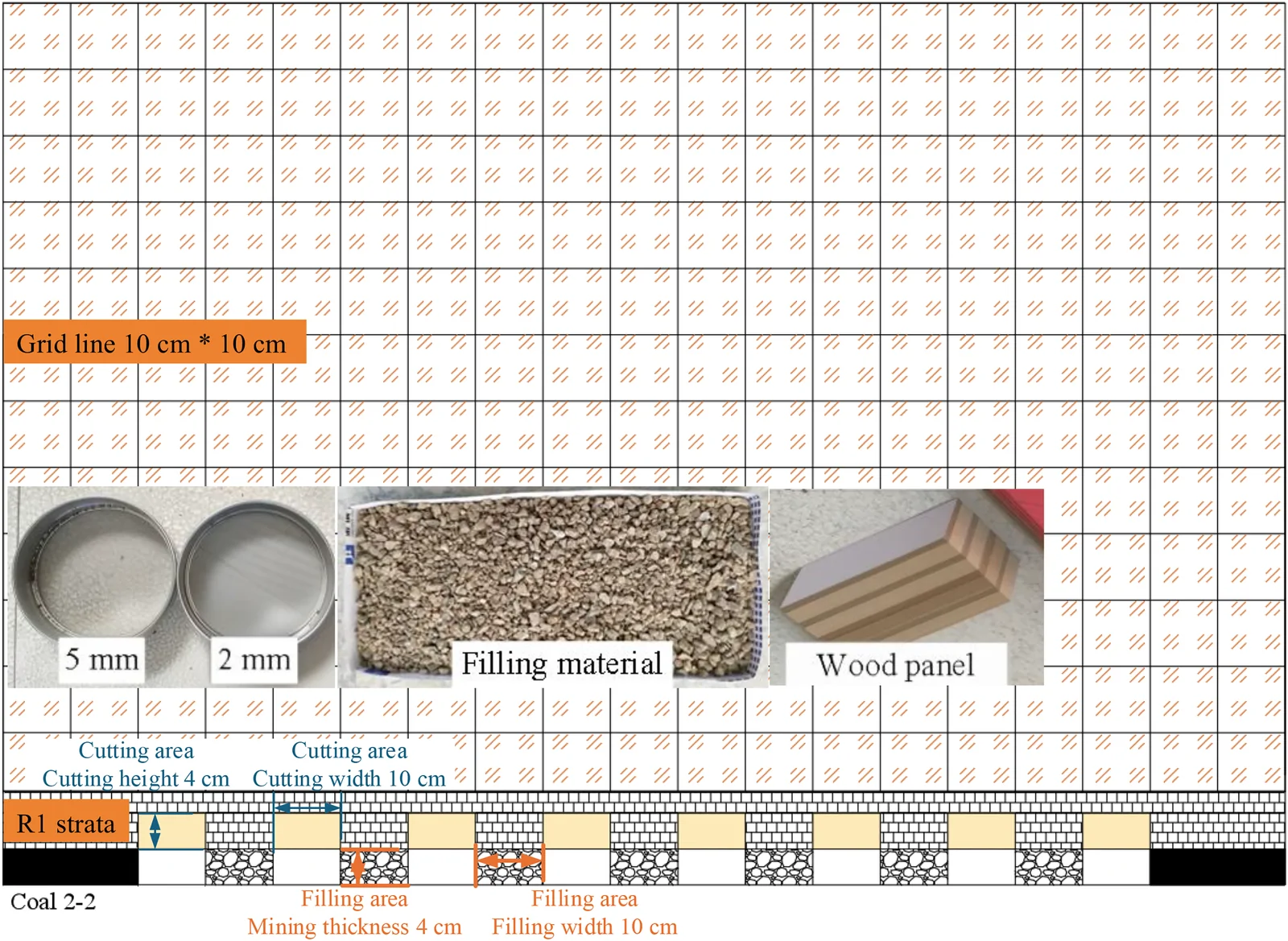
Similar simulation experiment scheme of Cut-and-fill mining. The diagram shows the model grid layout, test materials (filling particles and roof-cutting panels), and the dimensional design of cutting and filling zones at the model bottom, with a geometric similarity ratio of 1:100.

As shown in Fig 2, to facilitate the simulation of roof cutting, roof cutting wood boards were pre-embedded at the bottom of the R1 sandy mudstone to simulate the roof cutting stratum. The size of the roof cutting wood board is 22 cm × 10 cm × 3 cm, corresponding to simulating the roof cutting range with a thickness of 3 m and a width of 10 m in the field. The filling material is sand particles with a particle size of 2 ~ 5 mm, which is selected according to the geometric similarity ratio of 1:100. This particle‑size range matches the scaled gradation of on‑site broken coal gangue, reproducing the mechanical compaction characteristics of field gangue after size reduction by similarity criteria. which are put into paper boxes with a size of 22 cm × 10 cm × 4 cm to make filling strips, simulating the filling strips with a height of 4 m and a width of 10 m in the field. The experimental design mining range is 150 m, and a total of 8 roof cutting wood boards are pre-embedded.

In Cut-and-fill mining, the deformation characteristics of gangue pillar directly determine the effect of roof control, which is the core factor to ensure the stability of surrounding rock. In order to simulate the failure process of the stope when the filling ratio is insufficient, analyze the instability mechanism, and clarify the influence of the filling ratio, this experiment adopts a control design, and sets up two groups of parallel experiments of 100% filling ratio and 50% filling ratio, and clarifies the difference between the failure and instability of the stope under the low filling ratio through data comparison.

In order to facilitate roof cutting, a 22 cm × 10 cm × 4 cm top-cut plank (corresponding to a top-cut width of 10 cm and a thickness of 4 cm) was embedded at the bottom of R1 Sandy. The planks are removed after roof‑cutting and do not participate in stope load‑bearing, so only the geometric dimensions are similar, and the mechanical properties of the material are not considered.

The 100% filling ratio group simulates the ideal working condition and requires dense filling without voids. Combined with the geometric similarity ratio (actual coal breaking and cutting block 0.1 ~ 0.5 m), 1 ~ 5 mm sand was filled in a 22 cm × 10 cm × 4 cm paper box to simulate 4 m high and 10 m wide gangue pillars, and the filling ratio was strictly controlled to 100%.

In the 50% filling ratio group, foam paper was selected as the filling material. In order to ensure the rationality of the simulated material, the mechanical properties of foam paper were tested and analyzed by uniaxial compression pre-test. Based on the test results, the filling parameters were determined to simulate the compression characteristics of low filling ratio gangue. Combined with the working conditions of poor filling quality and serious gangue loss in the project, the filling ratio is set to 50%.

In this test scheme, 100% filling represents the ideal full‑support working condition for cut‑and‑fill mining in engineering practice. By contrast, 50% filling reproduces common on‑site adverse scenarios including incomplete filling and gangue loss. This two‑group controlled comparison test aims to reveal the differences in overburden failure and stope instability between ideal filling state and actual defective filling state, which carries clear practical engineering reference value.

The basic parameters of the two groups of tests are the same, and the filling laying, construction and displacement observation schemes are unified to eliminate the interference of irrelevant variables. After the construction is completed, the deformation of gangue pillar is observed synchronously, and the difference is compared and analyzed. The influence of insufficient filling ratio is clarified, which provides reliable test data for the analysis of stope instability mechanism.

### 2.3. Experimental analysis

It can be seen from Fig 3 (a) that after the 100% filling ratio Cut-and-fill mining is completed, there is a clear separation at the top of the R3 rock layer. Each gangue pillar has uniform force and reliable support, and there is no adjacent gangue pillar linkage instability, and the whole is in a stable support state.

**Fig 3:**
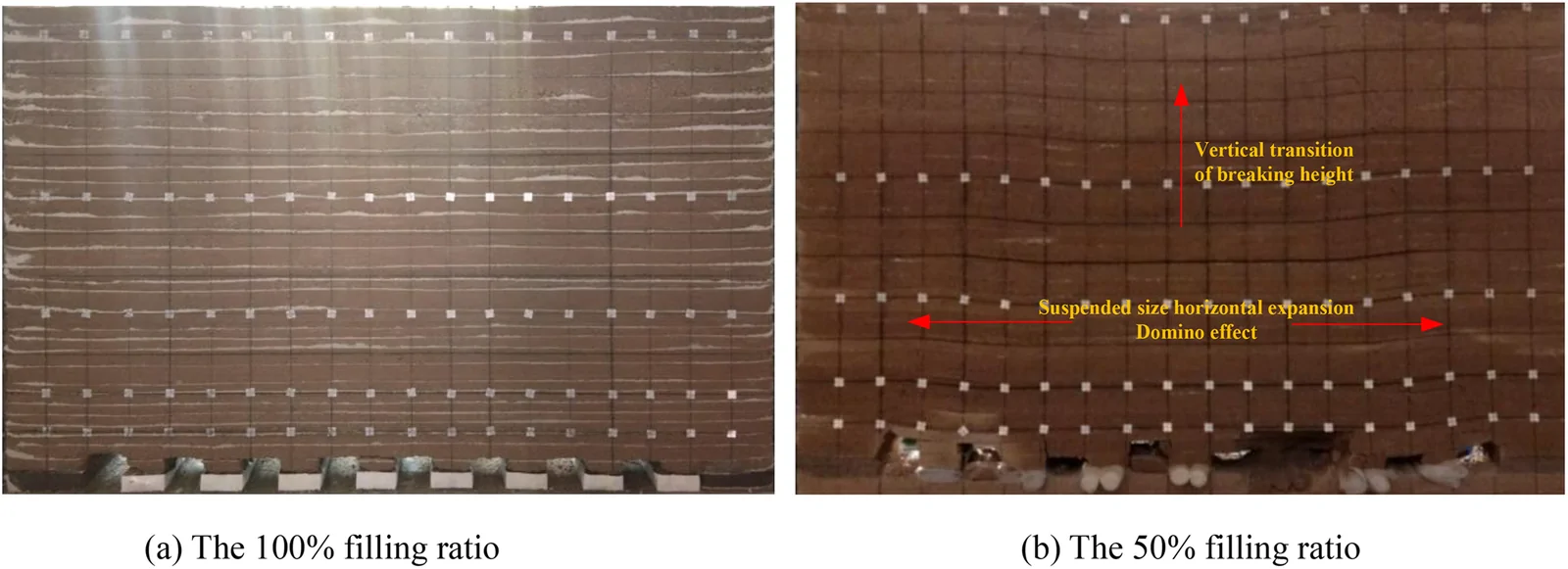
Overburden failure characteristics of Cut-and-fill mining. (a) 100% filling ratio. Overburden remains generally stable with only slight local separation; gangue pillars bear load uniformly without linked instability. (b) 50% filling ratio. Insufficient filling triggers domino-style chain failure of gangue pillars starting from the goaf center, resulting in key stratum fracture and severe overburden damage.

Through the analysis of similar simulation test, it can be seen that the gangue filling ratio reaches 100%, which can effectively limit the movement space and hanging size of the roof. The key stratum has not reached the size condition and movement space required for breaking. Therefore, the key stratum has not broken, and the gangue pillar support at this place is stable without instability.

After the end of the model Cut-and-fill mining in the 50% filling ratio group, the gangue pillar gradually compacted and deformed, and the roof movement process is shown in Fig 3 (b).

As shown in Fig 3 (b), the collapse range of the immediate roof is expanded, the crushing is serious, and the obvious separation layer appears at the bottom of R3 sandstone. With the further deformation of gangue pillar, R3 sandstone fractured, and the bottom of R6 medium grained cracked.

Due to the insufficient filling ratio, the roof above the gangue pillar near the middle of the goaf is severely damaged, and the supporting effect is lost. The original overburden load is transferred to the adjacent gangue pillar, causing the adjacent gangue pillar to be destroyed. The domino effect occurs in the gangue pillar system, which eventually leads to the fracture failure of the overlying key layer R6 medium grained pillar, and the fracture directly reaches the surface, resulting in severe surface subsidence. The insufficient filling ratio results in the severe failure of the Cut-and-fill mining overburden, which is similar to the mining by caving method.

It can be seen from Fig 4 (a) that in the 100% filling ratio group, the 110 m, 70 m and 40 m lines near the surface did not deform due to the filling of gangue pillars, the 10 m line showed volatility, and the 20 m line showed almost no volatility. Due to the friction hindrance effect of gangue pillar on the horizontal movement of overlying strata and the uniform support effect, the horizontal displacement is very small, showing a positive and negative alternating distribution. Due to the edge effect, the horizontal displacement of the measuring point near the coal pillar is too large.

**Fig 4:**
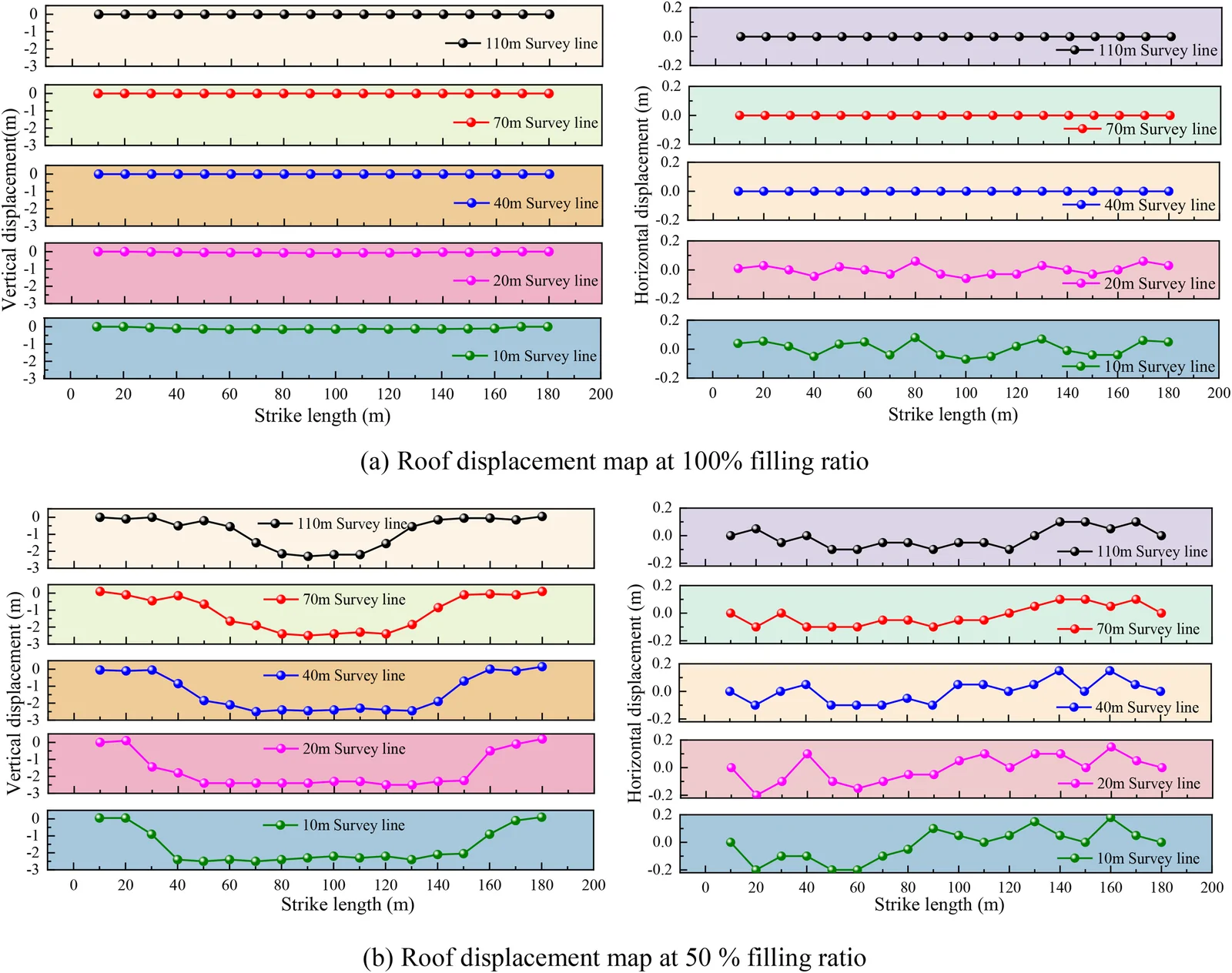
Roof displacement diagram of similar simulation test. (a) 100% filling ratio. Vertical (left) and horizontal (right) displacements along strike length at 10–110 m survey lines show minimal strata deformation under full filling. (b) 50% filling ratio. Vertical displacement reaches 2.19 m at the 110 m survey line with a symmetrical subsidence profile, while horizontal displacement remains limited.

It can be seen from Fig 4 (b) that after the failure of the 50% filling ratio group, the displacement of the rock layer is intensified, and the maximum vertical displacement is 2.19 m from the 110 m survey line of the coal seam. Because the main form of overburden movement is flat drop, the horizontal displacement changes little. The maximum horizontal displacement of the survey line 110 m away from the coal seam is 0.10 m. Although the model fails, compared with mining by caving method, Cut-and-fill mining still has a certain effect on controlling subsidence.

Through the analysis of similar simulation test, it can be seen that the filling ratio of gangue pillar is insufficient, and the roof movement space and hanging size are given at the same time. When the breaking size and movement space of the key stratum are satisfied at the same time, the key stratum will break, and the instability of the gangue pillar will occur. After the instability of a gangue pillar, the gangue pillars on both sides are rapidly destabilized, showing a domino effect.

## 3. Numerical simulation

### 3.1. Research method

#### 3.1.1. Selection of relevant parameters.

The Flatjoint model in PFC numerical software provides a macroscopic behavior of linear elastic constraints between particles and linear elastic friction states that allow partial damage. The continuous accumulation of damage to the contact surface leads to the sudden change of the unit from the fully constrained state to the completely unconstrained friction state, and finally the macroscopic cracks are formed in the model. Flatjoint contact simulates the relationship between the imaginary interfaces of two rigid particles, which is closer to reality in field applications. Therefore, the Flatjoint contact model is selected for the contact between particles in numerical simulation [34,35].

The closer the rock mass parameters used in numerical simulation match field rock‑mass properties, the more reliable the simulation results. However, in the actual process of establishing the model, it is necessary to simplify the engineering practice. In this paper, the simulated rock strata are simplified by referring to the general situation of coal mining area, the occurrence conditions of rock strata and the basic mechanical parameters of rock strata. There are 5 layers of rock that need to be checked after simplification, and the relevant parameters are shown in Table 2.

**Table 2:** Formation parameters of Coal Mine.

Lithology	Volume force/ kN/m^3^	Elastic modulus/ GPa	Compressive strength/ MPa	Tensile strength/ MPa	Internal friction angle/°	Cohesive force/ MPa
Conglomerate	20	1.30	11.90	1.10	31	1.1
Medium grained sandstone	22	2.83	21.23	1.9	33	1.9
Sandstone	24	3.35	28.43	2.1	33	2.1
Sandy mudstone	20	1.67	16.23	1.3	28	1.2
Coal seam	14	1.44	10.21	1.2	30	1.0

It gives the macroscopic mechanical parameters of five simplified lithologies, which serve as the calibration benchmarks for mesoscopic particle parameters in the numerical model.

Reference [34,35] pointed out that the loading rate had a relatively small effect on the tensile strength of the test block; in the uniaxial compression experiment, with the increase of loading rate, the uniaxial compression curve has a great difference after entering the failure stage. In the uniaxial compression process, when the loading rate is below 5 mm/s, although the stress peak of the test block is different in the failure stage, the yield limit of the two tests is basically the same. Therefore, the 5 mm/s loading rate is selected for simulation analysis in the subsequent parameter check. In the uniaxial tensile process, the loading rate of 0.2 mm/s was selected for simulation analysis.

The field model is large, and it is difficult to establish a model that is consistent with the particle size of the calibration block, so the size of the test block needs to be scaled. Reference [34] pointed out that when the mesoscopic parameters are checked, the size of 50 mm * 50 mm can take into account the calculation efficiency and calculation accuracy. At the same time, the literature [35] points out that the average radius of the particles is 600 mm, and the corresponding test block size is expanded to 30000 mm, which is basically the same as the uniaxial compressive strength and tensile strength when the average radius of the particles is 1 mm and the test block size is 50 mm. Therefore, the size of the test block is 19000 mm * 13400 mm. In the literature [36,37], it is pointed out that when the height L of the specimen and the particle radius R satisfy L/R > 60 in the standard specimen, the particle size has little effect on the calculation results. Therefore, the particle radius range is 300 mm ~ 450 mm when the calibration specimen is established.

#### 3.1.2. Rock mass parameter check.

Parameter verification is a repetitive activity. The author uses Python to perform automatic verification, automatically calculates the error of uniaxial compressive strength, uniaxial tensile strength and elastic modulus parameters, and gives the weight of 1/3 of the three parameter errors. When the single error is within 0.010 and the total error is within 0.010, the parameters are considered reasonable and the verification is stopped. This method can greatly reduce the check time and improve the accuracy of the check.

Taking the Conglomerate check as an example, the number of Conglomerate checks is 357 times. Among them, Run_ID is the number of checks, ErrTotal is the total error, ErrUCS is the uniaxial compressive strength error, ErrTS is the uniaxial tensile strength error, and ErrYMod is the elastic modulus error. When the total error condition is satisfied, ErrTotal is 0.009, ErrUCS is 0.003, ErrTS is 0.009, and ErrYMod is 0.010. When the error meets the conditions, the uniaxial compressive, uniaxial tensile failure modes and stress-strain curves of the test block are shown in Fig 5 (a) and (b), respectively.

**Fig 5:**
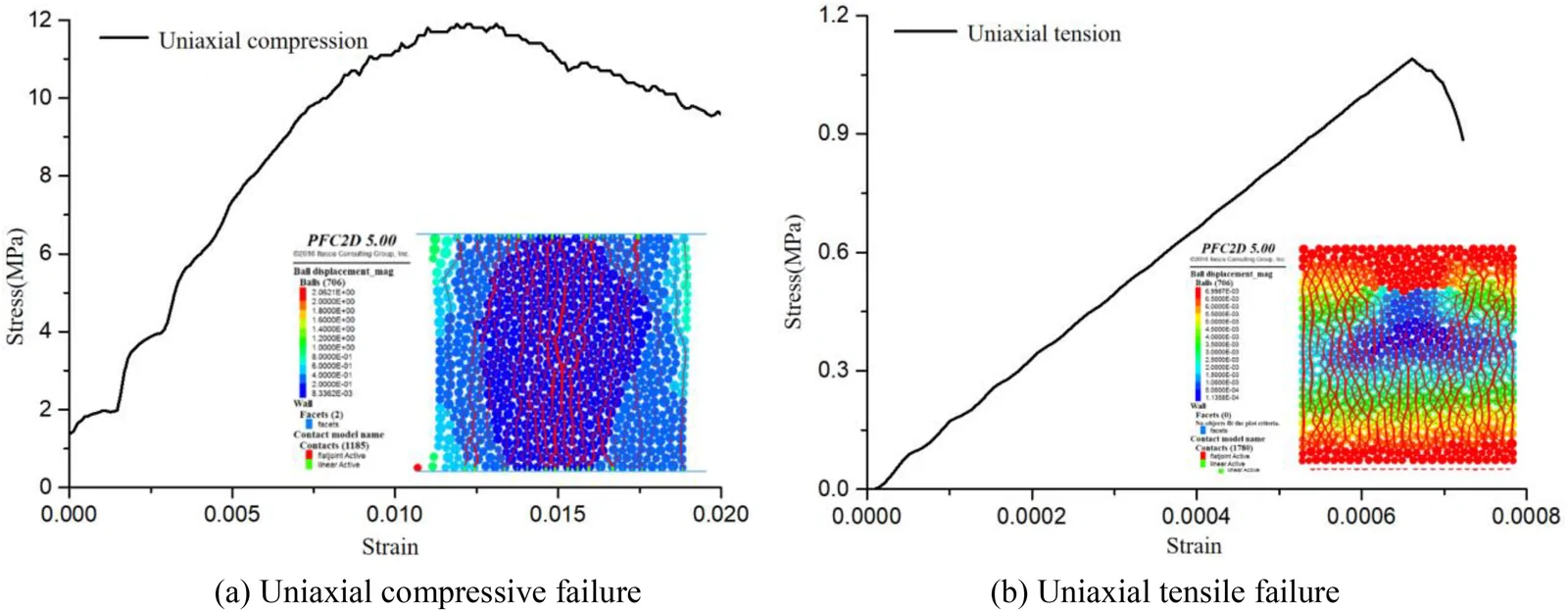
Parameter calibration of conglomerate test block. (a) Uniaxial compressive failure. Stress-strain curve and particle displacement cloud of the conglomerate specimen under uniaxial compression. (b) Uniaxial tensile failure. Stress-strain curve and particle displacement cloud of the conglomerate specimen under uniaxial tension.

The calculation results of the particle input meso-parameters and the macro-parameters based on the parameters in the numerical model when the corresponding strength of each rock layer is obtained by multiple debugging are shown in Table 3.

**Table 3:** Particle input parameters and test block output parameters.

Rock strata	Particle input parameters						Test block output parameters		
	fj_emod /GPa	fj_krat	fj_fric	fj_ten /MPa	fj_coh /MPa	fj_fa /°	Compressive strength /MPa	Tensile strength /MPa	Elastic modulus /GPa
Conglomerate	1.41	3.75	0.52	1.47	1.10	25.51	11.87	1.09	1.31
Medium grained sandstone	3.01	3.06	0.55	2.55	2.59	25.98	21.08	1.90	2.86
Sandstone	4.48	2.53	0.51	6.38	4.55	21.19	28.45	2.10	3.32
Sandy mudstone	1.86	3.73	0.59	1.77	1.61	27.16	16.04	1.31	1.66
Coal seam	1.54	3.44	0.45	1.88	0.85	23.66	10.35	1.19	1.42
Filling gangue	0.01	1.00	0.36	0.05	0.10	20.00	0.29	0.04	0.11

It presents the calibrated Flatjoint mesoscopic input parameters and corresponding macroscopic mechanical outputs for each rock stratum and filling gangue, with calibration errors within the allowable range.

#### 3.1.3. Determination of mining method.

In order to ensure the consistency of the results, the numerical model is consistent with the size of the similar model. The model is 190 m long and 134 m high. The height parameters of each rock layer in the model are shown in Table 1.

In the test of roof filling mining, 20 m protective coal pillars were left on both sides of the model, and the width of the middle mining section was 150 m. There were 8 roof cutting areas and 7 filling areas along the tendency of the working face. The width of roof cutting was 10 m, the height of roof cutting was 4 m, the width of filling was 10 m, and the height of filling was 4 m.

In the PFC modeling workflow, rock‑mass particles are generated first, followed by deleting particles within pre‑defined roof‑cutting zones to reproduce roof‑cutting operations. Then gangue‑filling particles are generated inside each designated filling region. Both simulation groups (100% and 50% filling ratio) adopt the identical particle‑generation algorithm, particle‑size distribution and meso‑mechanical parameters for gangue particles. Notably, the filling ratio is realised purely by tuning the target porosity within filling zones. Neither gangue particle strength nor particle size is altered, and no generated particles are deleted after generation. The filling ratio corresponds to the volume fraction of solid gangue particles occupying the total filling‑zone space. A low target porosity represents 100% dense filling; elevated target porosity yields 50% filling state, reproducing the on‑site incomplete‑filling scenario with gangue loss. Only the target porosity inside filling zones is adjusted to realise different filling‑ratio conditions. Such unified particle‑generation rules exclude differences originating from particle properties, ensuring result comparability between the two groups.

The mining technology consistent with that of similar models was adopted for this research. For both the 100% filling ratio condition and the 50% filling ratio condition, the PFC numerical simulation software was employed to set the calculation steps to 20000, 40000, 60000, 80000, 100000, 120000, 140000 and 160000 respectively. The displacement law and force chain distribution characteristics of the overlying strata at different calculation stages were systematically acquired, thus revealing the dynamic evolution process of overlying strata stability under the full filling condition.

### 3.2. Simulation analysis of 100% filling ratio

As shown in Fig 6, since the filling ratio reaches 100%, there is almost no change in the rock strata within the main roof during the whole calculation process, and there is no obvious subsidence phenomenon. The rock strata are closely combined without any signs of separation. The main key stratum R6 medium grained bedding always maintains a complete and intact state, and its top always maintains a stable and dense closed force chain structure.

**Fig 6:**
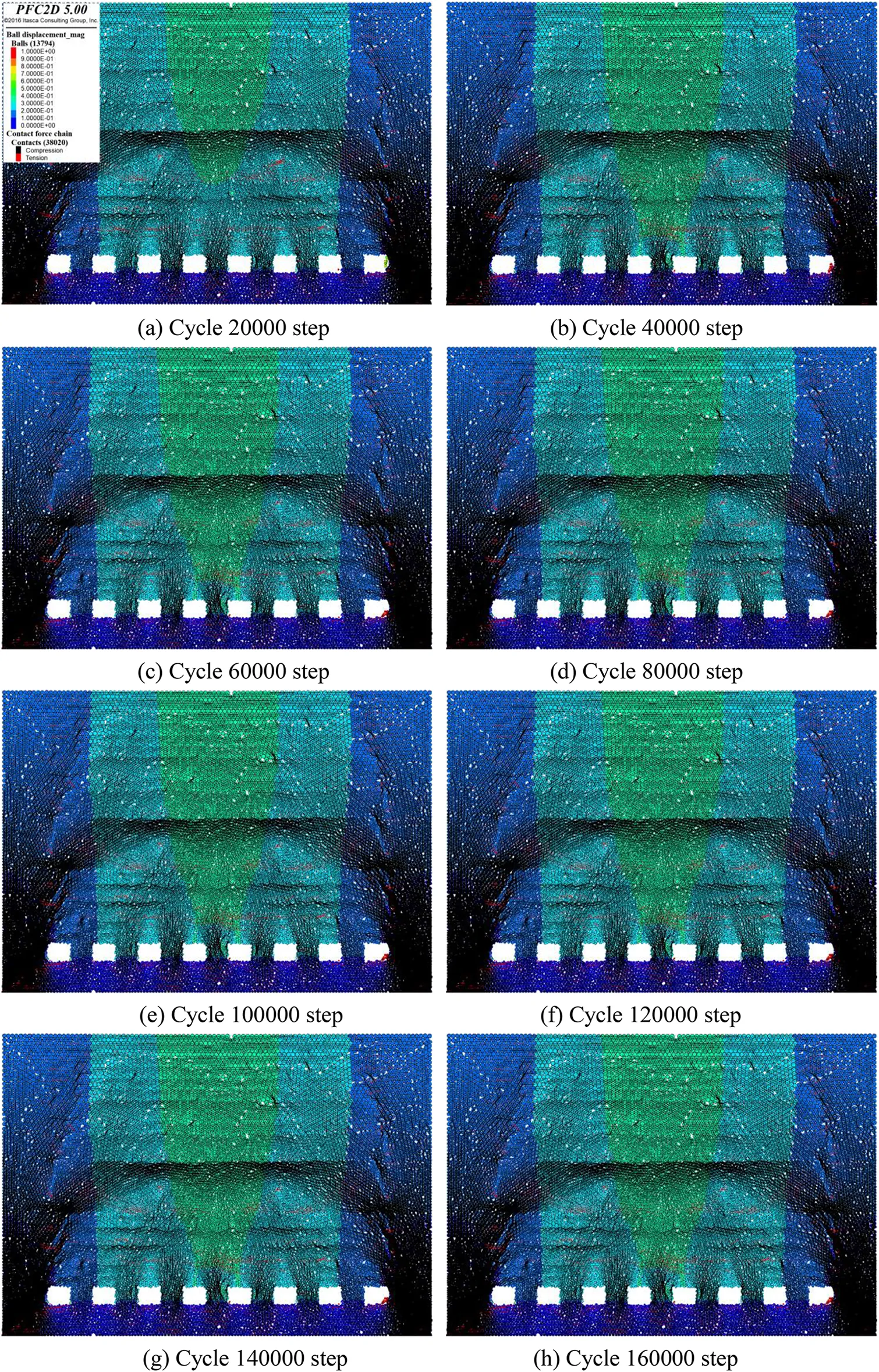
The failure process of overlying rock in Cut-and-fill mining with different cycle steps. PFC simulation shows that under full filling, only scattered micro-cracks develop locally in the roof. The force chain remains dense and regular, and the main key stratum stays intact with no obvious subsidence throughout the calculation.

As shown in Fig 6 (a), when the calculation is carried out to 20,000 steps, only 1080 sporadic cracks appear in the roof rock layer, mainly concentrated in the local area on the top and both sides of the roof rock pillar. The number of cracks is small, the scale is small, and no penetrating damage is formed. It only shows the characteristics of small local stress release and does not affect the stability of the overall structure.

As shown in Fig 6 (b), as the calculation progresses to 40000 steps, the number of cracks increases to 1150. Although there is a slight increase, it is still dominated by locally dispersed small cracks, and there is no large-scale expansion or connection of cracks. There are only a small number of fine cracks in the contact area between the gangue pillar and the roof, and the overall force chain structure remains dense and regular.

As shown in Fig 6 (c)~(h), when the calculation is carried out to 60000 steps, the number of cracks only increases to 1152, and the growth rate is extremely weak. All cracks are limited to a very small range around gangue pillar, and there is no obvious sign of expansion or penetration.

Because the filling spacing of Cut-and-fill mining is equal and the distance is small, and the gangue pillar filling ratio is full and the support strength is sufficient, the roof is always flat and stable, and does not show any bending deformation and caving trend. The stress of gangue pillar in each area is uniform and balanced, and there is no local stress concentration. The distribution of force chain is always regular and dense, and there is no sparse, slender and bearing phenomenon in the whole process. Each gangue pillar always bears stably and gives full play to the support efficiency. The overall structure remains stable, and there are no signs of crack penetration, fracture and instability.

### 3.3. Simulation analysis of 50% filling ratio

In order to obtain the failure state of overburden rock at different stages under the condition of insufficient filling ratio (50%), PFC numerical simulation software is used to obtain the displacement and force chain distribution characteristics of overburden rock at different calculation steps by controlling the number of calculation steps, and further reveal the evolution process of Cut-and-fill mining due to insufficient filling ratio.

As shown in Fig 7 (a), due to the insufficient filling ratio, the main roof rock layer sinks sharply after 20000 steps of calculation, and obvious separation occurs under R6 medium grained bedding. Relying on its main key stratum bearing function, a closed force chain structure is formed at the top. Affected by the uniform and small spacing of Cut-and-fill mining, the roof subsidence is characterized by mining by caving method. In the middle of the goaf, the stress is concentrated, forming a dense force chain and bearing first. At this time, the number of fissures is 2221, which is scattered at the edge of the goaf.

**Fig 7:**
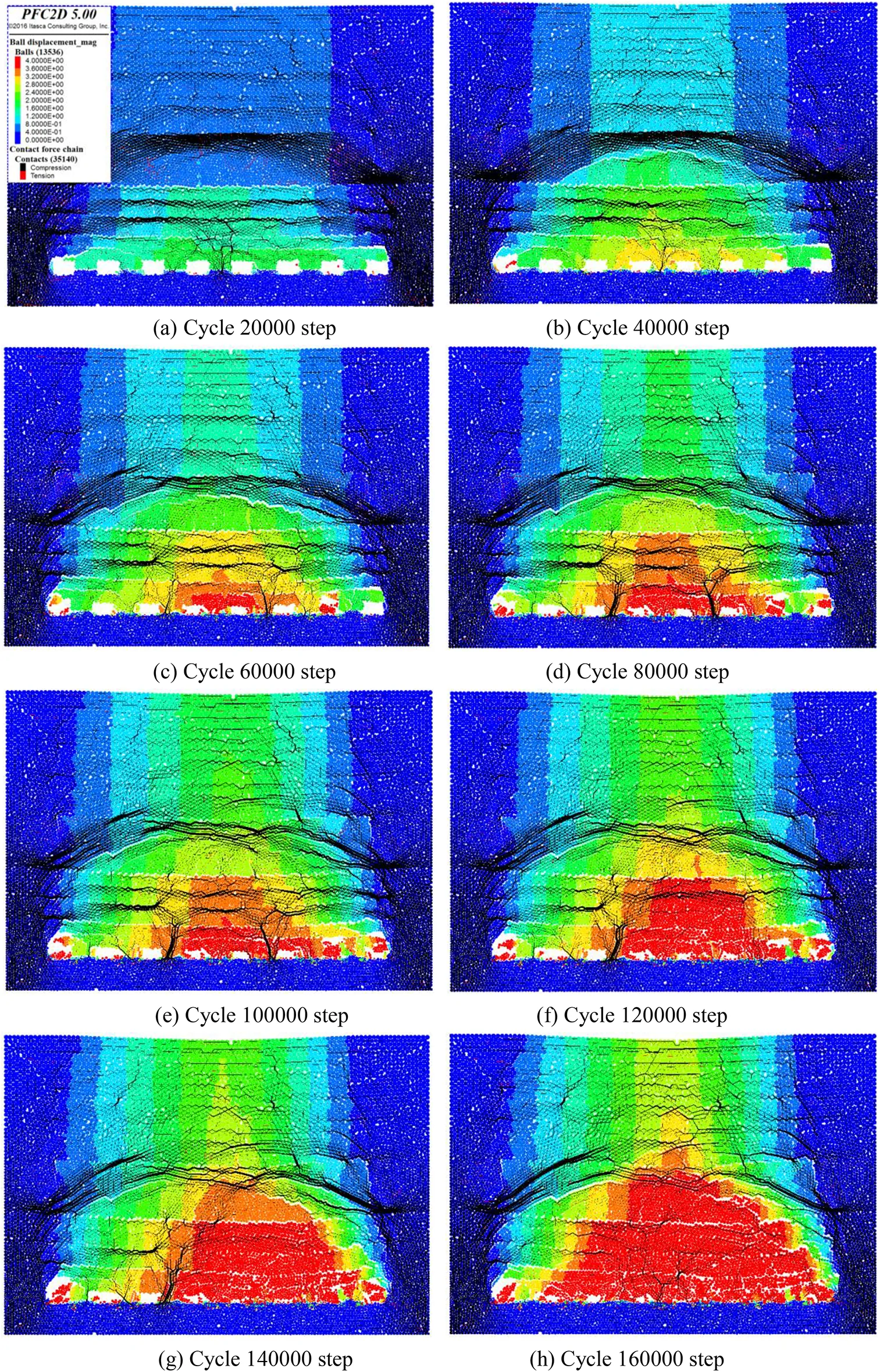
The failure process of overlying rock in Cut-and-fill mining with different cycle steps. PFC simulation shows that under insufficient filling, roof bending intensifies gradually with calculation steps. Cracks propagate and interconnect; the central gangue pillar fails first, and the force chain shifts to both sides, inducing progressive chain instability.

As shown in Fig 7 (b), with the increase of the number of calculation steps, the bending deformation of the roof increases, the bottom of the R6 medium grained crushed rock is destroyed, the top force chain is sparse, and the main key stratum is attenuated. There are 3496 fractures in total, which extend to the middle of the goaf and are locally connected. The deformation of the middle gangue pillar is significant, the force chain is weakened, and the bearing capacity decreases rapidly.

As shown in Fig 7 (c)~(d), the number of calculation steps is further increased, the damage range of R6 medium grained sandstone is expanded to both ends of the fracture, the top force chain continues to be sparse, and the main key stratum bearing capacity is sharply reduced. At this time, the number of fractures increased to 5022, forming local fracture networks. The middle gangue pillar force chain almost disappears, the adjacent gangue pillar force chain develops and becomes thicker, and the bearing body transfers to both sides. Due to the maximum displacement gradient difference at both ends of the goaf, the roof changes from continuous bending to caving.

As shown in Fig 7 (e)~(f), with the increase of the number of calculation steps, the number of fractures reaches 6609. Under the action of rock hinges, only residual bearing capacity remains. The force chain structure at the top not only becomes very sparse and slender, but also presents new characteristics of discontinuity. The force chain structure of gangue pillar on both sides of the middle of the goaf began to become thinner and thinner, until the force chain structure on the side close to the roof caving disappeared.

As shown in Fig 7 (g)~(h), when the number of fractures increases from 7456 to 8235, the discontinuity of the force chain structure at the top of the R6 medium grained fractured rock is enhanced. All the gangue pillars force chain structures located on the left side of the middle of the goaf also disappeared, and the sparse force chain structure appeared in the middle of the whole goaf, showing the characteristics of residual support.

Through numerical simulation analysis, it can be seen that when the filling ratio is insufficient, the roof will be unstable, and the instability process generally shows an approximately continuous process. Among them, the deflection of the roof in the middle of the goaf is the largest, and the displacement difference is the largest at both ends of the goaf. The two roofs are the first to be damaged. The gangue pillar in the middle of the goaf has the most obvious force, which first shows the bearing effect and the first damage. Therefore, in the process of design and practice of Cut-and-fill mining, it is necessary to strengthen the gangue pillars in the middle and both ends of the goaf.

Through the evolution law of force chain in different mining stages, it can be obtained that the stability of gangue pillar in the middle of goaf is the premise of the stability of the whole stope. With the instability of gangue pillar in the middle of goaf, the center of force chain gradually transfers to both sides, which may lead to the chain failure of gangue pillar. The load transfer caused by the local instability of gangue pillar is the essential reason for the chain failure of gangue pillar.

## 4. Discussion

Fig 8 is the force chain network diagram of Cut-and-fill mining after stable mining when the filling ratio is relatively high, which is obtained by PFC numerical simulation. The so-called force chain is the interaction between particles through contact, and the thickness of the force chain represents the size of the contact force. The network formed by particles contacting with each other becomes a force chain network.

**Fig 8:**
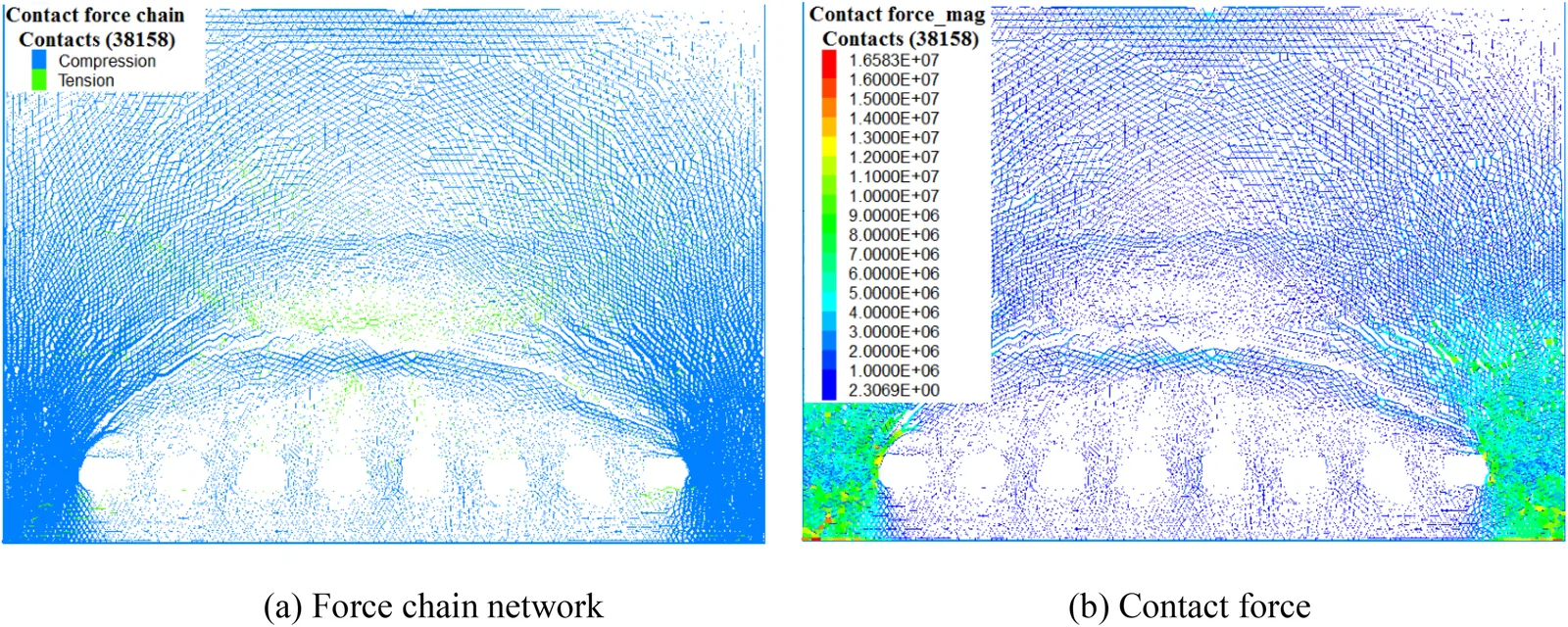
Contact force and force chain after Cut-and-fill mining. (a) Force chain network. A closed arch-shaped force chain forms at the bottom of the main key stratum, and small closed force arches exist between adjacent gangue pillars. (b) Contact force magnitude. The force magnitude distribution reflects the load-bearing characteristics of the gangue pillar-roof support system.

From Fig 8, it can be seen that after the Cut-and-fill mining is stable, the force chain network at the bottom of the medium grained cemented (main stratum) in the roof area forms a closed arch, and a small closed arch is also formed between the adjacent filling areas of the goaf. This phenomenon is very similar to the pressure arch proposed by German scholars Huck and Gillitzell [38]. In the mined-out area, the granite column together forms a force chain network supporting the medium grained cemented, which is the physical and mechanical basis for controlling subsidence and reducing pressure in Cut-and-fill mining.

Combined with the results of similar material simulation test and the surrounding rock control theory of coal mine stope, the overburden stress above the roof stope of Cut-and-fill mining is characterized by ‘composite stress arch’. As shown in Fig 9, after the end of mining, a small range of regular ‘internal stress arch’ is formed between the gangue pillars in the adjacent filling area, and a large range of ‘external stress arch’ is formed at the lateral edges on both sides of the working face. The low roof of the cutting area is affected by mining damage, forming fracture arch [38–40]. Corresponding to the three overburden failure spatial structures of Cut-and-fill mining, the ‘composite stress arch’ also has a corresponding evolution.

**Fig 9:**
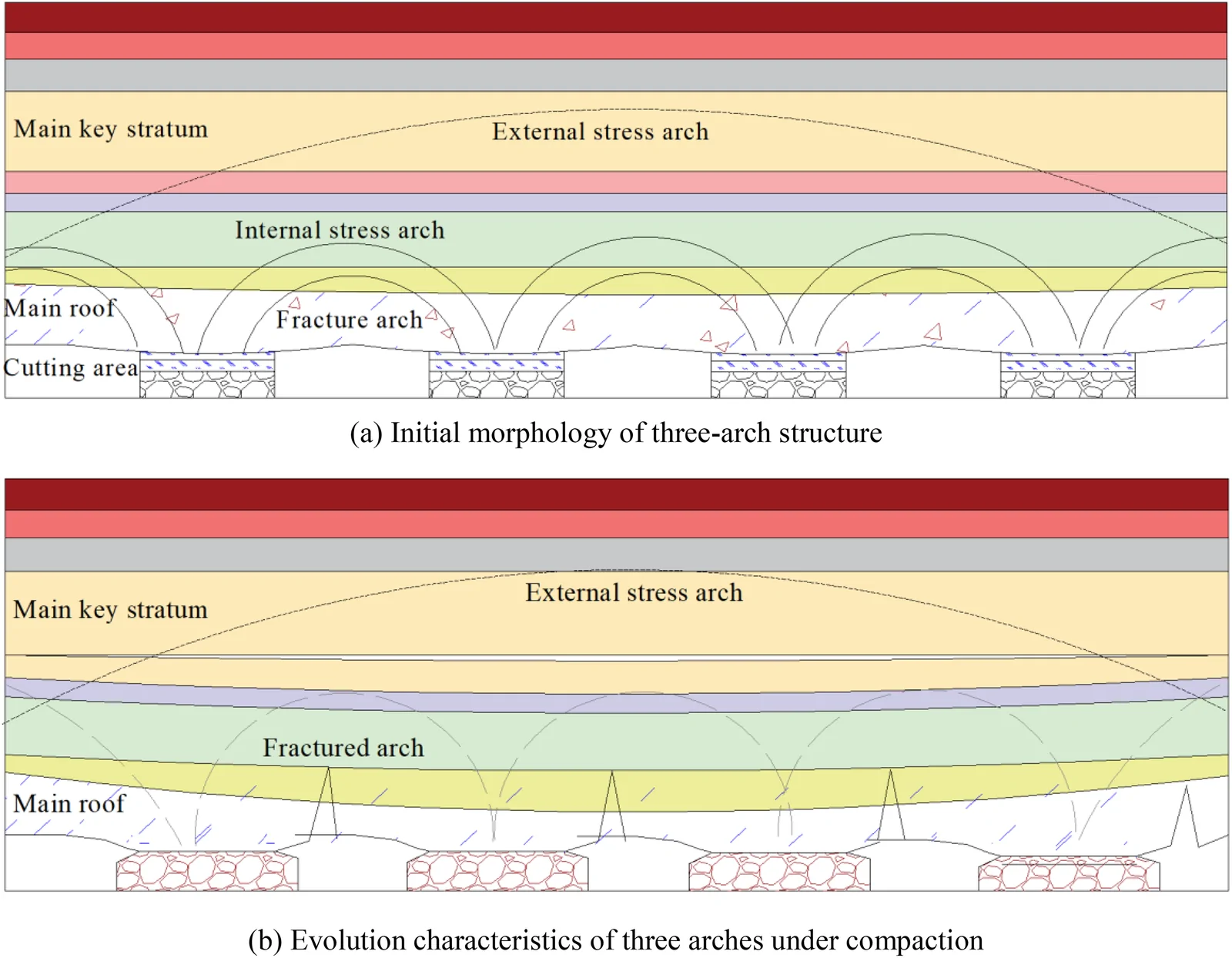
Schematic diagram of the ‘composite stress arch’ model for Cut-and-fill mining. (a) Initial morphology of three-arch structure. The composite system consists of external stress arch, internal stress arch and fracture arch after mining. (b) Evolution characteristics of three arches under compaction. With gangue pillar compaction, the fracture arch expands upward and merges with the internal stress arch; further upward propagation will collapse the outer stress arch system.

The ‘external stress arch’ is a half-space annular shell, as shown in Fig 9 (a). Both ends of the arch foundation are located in the stress-increasing area of the coal body on the side of the lateral goaf, and the vault is located in the high-level hard roof. The ‘fracture arch’ is like a semi-ellipsoid in the overlying strata of the stope. The arch foundation is located at the edge of the gangue pillar on both sides. The ‘internal stress arch’ is also a half-space annular shell, wrapping the space of ‘fracture arch’, and ‘internal stress arch’ and ‘external stress arch’ jointly bear the rock load from it to the surface.

As shown in Fig 9 (b), With the compaction of gangue pillars, the arch base of the fracture arch gradually expands to the center of gangue pillar on both sides, and gradually merges with ‘internal stress arch’, corresponding to the fracture of the low main roof. If the fractured arch continues to develop upward, the ‘fractured arch’ is connected to each other, the ‘internal stress arch’ will disappear, the overburden damage will increase, the ‘fractured arch’ continues to develop upward, the main key stratum is broken, the ‘external stress arch’ will disappear, and the overburden damage will affect the surface.

From the theoretical perspective of voussoir‑beam and stress‑arch theory, the overburden load is redistributed after roof cutting and filling mining. Part of the overlying strata weight is borne by the coal‑body boundary on both sides through the external stress arch effect, and the residual load transfers downward to each gangue‑pillar support unit. The vertical load acting on a single gangue‑pillar can be expressed as:


$$\begin{array}{c} P_{g}=k_{a}\cdot \gamma \cdot H\cdot B-\sigma_{arch} \end{array}$$

Where ${\text{P}}_{\text{g}}$ is vertical load on gangue pillar; $K_{a}$ is load‑redistribution coefficient caused by stress‑arch effect; γ is average unit weight of overburden; H is overburden burial depth; B is the effective bearing width of gangue pillar; $\sigma_{arch}$ is the load shared by external stress‑arch transfer.

When the bearing capacity of the middle gangue pillar satisfies $P_{g}>R_{g}$ is ultimate bearing strength of gangue pillar), the middle pillar yields and fails. After local failure occurs, the original load borne by the failed pillar is transferred to adjacent gangue pillars, increasing the load of neighbouring pillars. When the transferred superimposed load exceeds their ultimate bearing capacity successively, domino‑style chain instability will be triggered. This theoretical criterion explains why the middle gangue pillar preferentially fails under low filling‑ratio conditions observed in physical and numerical simulations. The evolution of composite three‑arch structure is essentially the continuous adjustment process of this load‑redistribution system under gangue‑pillar compaction and damage.

Comparing with previous related studies, many existing filling‑control investigations mainly focus on vertical compaction characteristics and critical compaction indexes of backfill bodies [21,27‑29]. Consistent with those studies, our work also confirms that insufficient filling condition will trigger key‑stratum fracture and aggravated overburden damage. However, different from previous studies that rarely considered horizontal filling‑ratio gradients and gangue‑pillar chain‑instability behaviour, this paper further reveals the domino‑style progressive failure originating from middle goaf gangue pillars, and proposes the composite three‑arch stress‑evolution model for cut‑and‑fill mining. It provides a supplementary understanding for strata‑control theory under gangue‑pillar cut‑and‑fill mining conditions.

There are still several limitations in this work. First, the physical similar‑material model follows a 1:100 geometric similarity ratio; scale‑effect deviations may exist between laboratory model responses and full‑scale field engineering conditions. Second, in PFC numerical simulation, several adjacent similar lithostratigraphic units are merged to reduce calculation cost, which brings certain simplification deviation compared with real complex geological strata. Third, the critical filling‑ratio threshold (70%‑90%) obtained in this paper is valid only for the geological background of the Ordos 2‑2 upper coal seam; the threshold value will shift under different burial depth, rock‑mass strength and stratum combination conditions. In future research, more field monitoring datasets will be collected to calibrate numerical parameters, and multi‑field‑condition numerical groups will be carried out to obtain a more generalized critical‑filling‑ratio evaluation method.

## 5. Conclusion

(1) The filling ratio is the dominant factor governing overburden and stope stability. Full (100%) filling suppresses roof displacement and key-stratum breakage, achieving reliable gangue-pillar bearing and effective overburden control. In contrast, 50% insufficient filling triggers key-stratum fracture and domino-style chain failure initiated by central goaf gangue pillars, further producing severe overburden destruction and large-magnitude surface subsidence.
(2) At 100% filling ratio, overlying rock maintains microscopic stability characterized by scattered non-penetrating micro-fractures, dense regular force chains and uniform loading on gangue pillars. Under 50% filling ratio, micro-fractures proliferate and interconnect into local fracture networks, inducing key-stratum fracture. Micro-scale force-chain-fracture evolution demonstrates that the stability of central gangue pillars determines overall stope safety, and local pillar instability shifts the force-chain center and triggers progressive chain failure.
(3) PFC simulation outcomes verify that Cut-and-fill mining produces a composite three-arch stress structure consisting of internal stress arch, fracture arch and external stress arch. The compaction-driven evolution of this composite structure governs overburden damage and surface subsidence. With progressive compaction of gangue pillars, the arch base of the fracture arch expands and merges with the internal stress arch. Sustained upward propagation of the fracture arch sequentially collapses the inner and outer stress-arch systems and gives rise to through-going overburden damage extending toward the ground surface. This three-arch evolutionary model clarifies stress transfer and rock-fracture laws for Cut-and-fill mining. Accordingly, field engineering design should implement reinforcement measures for central and end gangue pillars.
