# Supplementary material for: Study on the fracture characteristics controlled by filling ratio in cut-and-fill mining
Source: PLoS One. 2026 Oct 16;21(10):e0354444. doi: 10.1371/journal.pone.0354444 (PMC13674729; doi:10.1371/journal.pone.0354444)
Supplement: Supplementary file 1 [file pone.0354444.s002.docx]

We sincerely appreciate the editor‑in‑charge and the three reviewers for devoting their time and energy to reviewing our manuscript Study on the Fracture Characteristics Controlled by Filling Ratio in Cut‑and‑fill Mining. The insightful and detailed comments are of great value for polishing the academic content, experimental description, figure formatting and language expression of this work. We have responded to every concern item‑by‑item and implemented corresponding modifications in the revised manuscript, with revision marks provided. We would be grateful if you could consider our revised manuscript for publication.

Reviewer 1:

We thank the anonymous reviewer for the thorough review and thoughtful feedback on our manuscript Study on the Fracture Characteristics Controlled by Filling Ratio in Cut‑and‑fill Mining. All comments have been fully considered and addressed in the revised manuscript. The following presents the point‑by‑point responses to the reviewers’ comments.

1. The abstract covers rich content but is slightly stacked with detailed descriptions. It is suggested to appropriately streamline secondary process descriptions, further highlight core conclusions and key regulatory mechanisms, and improve the conciseness and readability of the abstract.

**Response:** Thank you for this constructive comment. We have revised the abstract accordingly. We removed redundant procedural descriptions of experimental‑simulation phenomena, condensed repetitive micro‑crack evolution narratives, and emphasized the core controlling mechanism of filling ratio, the composite three‑arch structural essence, and practical engineering suggestions. Specific mark‑ups including before‑and‑after comparisons are presented below. The revised abstract enhances logical compactness and highlights major findings without losing key information.

2. The literature review in the introduction is relatively comprehensive. It is suggested to add a separate paragraph at the end of the introduction to clearly condense the specific innovations of this work and the research gaps it fills, so that readers can quickly grasp the incremental contribution of the paper.

**Response:** Thank you for this valuable suggestion. Following your advice, we have added an independent stand‑alone paragraph at the end of the Introduction section (before the start of Section 2 Similar simulation. This newly‑added paragraph explicitly summarizes the existing research gaps and three core innovations of this study, highlighting the incremental academic contributions of the present work.

3. Please check and unify the coordinate axis labels and units of Fig.4 to ensure the consistency of marking specifications across the figure; Please check and correct the coordinate axis information of Fig.5 to ensure the accuracy of physical quantity names and unit marks; Appropriately improve the resolution of some figures and optimize the clarity of local labels to meet the standard requirements of journal publication.

**Response:** Thank you for your careful review and valuable suggestions on figure formatting. We have comprehensively revised Figures 4~5 as requested.

4. Please check the consistency of core data and conclusion expression between the abstract and the conclusion section, unify the description of key conclusions, and avoid expression differences, so as to enhance the logical rigor of the full text.

**Response:** Thank you for this important comment. We have carefully compared the revised abstract and the Conclusions section of the manuscript. We identified several inconsistent descriptive expressions for core findings between abstract and conclusions. We revised the Conclusions section to align terminology, failure‑mechanism description and structural‑model statement with the updated abstract. We unified key statements regarding filling‑ratio effect, gangue‑pillar chain‑instability mechanism and composite three‑arch structure.

5. It is suggested to carry out a full-text polish on the English writing, correct a small number of unidiomatic collocations and minor grammatical problems, and further improve the preciseness and academic standard of the language.

**Response:** Thank you for your careful comment on English language usage. We have performed careful full‑text polishing for the manuscript. Minor unidiomatic collocations, grammatical flaws, inconsistent terminology and awkward sentence structures have been revised.

6. Please uniformly sort out the reference citation format in the text and the list format at the end of the paper.

**Response:** Thank you very much for your careful review and valuable suggestion. We have comprehensively sorted out and standardized the in‑text citation format and end‑paper reference list format in the revised manuscript.

Reviewer 2:

We thank the anonymous reviewer for the thorough review and thoughtful feedback on our manuscript Study on the Fracture Characteristics Controlled by Filling Ratio in Cut‑and‑fill Mining. All comments have been fully considered and addressed in the revised manuscript. The following presents the point‑by‑point responses to the reviewers’ comments.

1. Briefly supplement the core innovation difference between this paper and existing research. Existing studies mostly focus on vertical compaction filling rate, while this paper systematically reveals the spatial-temporal fracture evolution of overburden and domino instability mechanism of gangue pillars under different horizontal filling ratios, add 1–2 short sentences to highlight this novelty.

**Response:** Thank you for your valuable comment. Following your suggestion, we have added two concise sentences in the newly‑added innovation paragraph at the end of Introduction (after original introductory content, before Section 2 Similar simulation). to highlight the core novelty difference from previous studies. We emphasize that most existing investigations focus on vertical compaction‑related filling rate, whereas this work addresses horizontal filling ratio and further reveals overburden spatiotemporal fracture evolution and the domino‑style gangue‑pillar instability mechanism.

2. When citing literatures about filling rate and strata control, briefly explain the theoretical basis of using composite stress arch theory to analyze gangue pillar load transfer in this paper, lay a clear foreshadowing for the subsequent theoretical model part.

**Response:** Thank you for your constructive comment. We have supplemented a short explanatory paragraph in the literature‑review segment of Section 1 Introduction, right after reviewing existing filling‑rate‑related studies. We briefly interpret the theoretical basis for adopting composite stress‑arch theory to analyse gangue‑pillar load transfer, and clarify why this arch‑based framework is suitable for cut‑and‑fill mining. This revision lays explicit textual foreshadowing for the subsequent composite three‑arch model presented in the Discussion section.

3. The basis for setting two working conditions (100% and 50% filling ratio) needs a brief supplementary explanation. Please add a small paragraph to illustrate that 100% filling represents the ideal full support working condition in engineering, while 50% filling corresponds to the common incomplete filling, gangue loss scene on site, and clarify the engineering comparative significance of the two groups of tests.

**Response:** Thank you for this valuable suggestion. We have added a short explanatory paragraph in Section 2.2 Experimental design, right after introducing the two parallel‑experiment schemes (100% and 50% filling ratio). The added text clarifies the practical engineering background of the two test conditions: 100 % filling stands for the ideal full‑support condition, whereas 50 % filling simulates frequent on‑site scenarios of incomplete filling and gangue loss. Meanwhile, we state the comparative engineering significance of this two‑group controlled test.

4. Briefly supplement the consideration of selecting 2–5 mm sand as filling aggregate in the physical model, and explain the similarity matching relationship between this particle size and on-site gangue gradation.

**Response:** Thank you for your constructive comment. We have supplemented a short explanatory sentence segment in Section 2.2 Experimental design, where the filling‑material description is located. We briefly illustrate the rationality for selecting 2‑5 mm sand as filling aggregate, and clarify the geometric‑similarity‑based matching relationship between this model particle size and the gradation of real‑site gangue.

5. Supplement a brief description of the particle filling and modeling operation steps in PFC, and briefly explain how the unified particle generation method ensures the comparability of 100% and 50% filling ratio simulation results.

**Response:** Thank you for your valuable suggestion. We have supplemented brief descriptions of particle‑filling workflow and modeling operation steps in Section 3.1.3 Determination of mining method. We also explained that the two groups adopt identical particle‑generation algorithm, particle size distribution and initial particle parameters; only the particle‑filling porosity is adjusted to realise 100 % and 50 % filling‑ratio conditions. This unified particle‑generation scheme eliminates particle‑related confounding variables and guarantees the comparability between the two simulation groups.

6. Uniformly standardize the captions of all figures (Fig.1–Fig.17, unify capitalization and punctuation rules; check redundant spaces and misaligned labels in SEM/PFC cloud map annotations, trim irregular layout marks.

**Response:** Thank you for your careful suggestions on figure formatting. We have comprehensively standardized all figure captions from Fig.1 to Fig.17 following PLOS ONE journal style requirements. We unified capitalization, sentence‑case writing style and terminal punctuation for each figure caption.

7. Carefully scan the whole text for typos, redundant spaces and non-standard abbreviations. If professional abbreviations appear for the first time, complete the full name; correct minor spelling errors to improve readability.

**Response:** Thank you for this careful proof‑reading suggestion. We have performed a full‑text manual inspection to identify typos, redundant blank spaces, and non‑standard abbreviations. For discipline‑specific abbreviations, the full complete name is supplemented at their first‑time occurrence in the manuscript. Minor spelling errors, run‑on compound‑word mistakes, extra blank characters and inconsistent terminology are corrected.

8. Briefly supplement the engineering application guidance value of the symmetrical load distribution law of gangue pillars, connect the theoretical calculation conclusion with the on-site layout optimization of middle and end gangue pillars.

**Response:** Thank you for this constructive comment. We have supplemented a short explanatory paragraph at the end of Section 4 Discussion. We elaborate the engineering guiding significance of the symmetrical load‑distribution law of gangue pillars under cut‑and‑fill mining. We further link the theoretical findings regarding load transfer and chain‑instability risk to on‑site layout and reinforcement optimization for middle and end gangue pillars.

Reviewer 3:

We thank the anonymous reviewer for the thorough review and thoughtful feedback on our manuscript Study on the Fracture Characteristics Controlled by Filling Ratio in Cut‑and‑fill Mining. All comments have been fully considered and addressed in the revised manuscript. The following presents the point‑by‑point responses to the reviewers’ comments.

1. The references are not cited in accordance with the manuscript formatting requirements. Please reasonably adjust the sequential order of the references.

**Response:** Thank you very much for pointing out this problem. We have comprehensively re‑checked the in‑text citation positions and the reference list of the manuscript.

2. The stratigraphic overview in Table 1 does not correspond to the information in the coal-rock stratum histogram of Fig. 1, and the thicknesses of each coal and rock stratum are inconsistent.

**Response:** Thank you for pointing out the inconsistency between Table 1 and the comprehensive strata histogram in Fig. 1. We have carefully cross‑checked the original borehole geological data. Table 1 retains the original borehole‑measured stratigraphic thickness data as the authoritative source dataset. We have redrawn the comprehensive strata histogram graphic inside Fig. 1, rescaling the vertical thickness of each stratum bar strictly according to each layer’s thickness value given in Table 1. The lithological sequence, stratum serial number and thickness values in Fig. 1 are now fully consistent with Table 1.

3. The parameters used in the numerical simulation tests in Table 2 do not correspond to those in Table 1. It is recommended to carefully check and revise them.

**Response:** Thank you for pointing out the parameter inconsistency between Table 1 and Table 2. Table 1 presents original borehole‑measured stratigraphic parameters of the engineering site. Table 2 is the simplified rock‑mass parameter set adopted for PFC numerical simulation, where partial lithologies from Table 1 are merged and simplified for calculation efficiency. We have re‑examined the lithology merging logic and mechanical‑parameter values. We have revised Table 2, making each lithological group’s mechanical parameters correspond reasonably to the weighted average of the matching original borehole strata in Table 1.

4. The expression of the unit weight unit in the tables is incorrect. The correct format should be “kN/m^3^ or kN·m⁻^3^”, rather than “kN.m^3^”.

**Response:** Thank you for pointing out the wrong format of unit‑weight symbols in tables. We have corrected the erroneous unit symbol kN.m^3^ to the standard format kN/m^3^ in Table 1 and Table 2.

5. The paper only sets two filling comparison groups (100% and 50%, lacking intermediate gradients such as 70% and 90%. This may fail to properly reveal the quantitative relationship between filling ratio and overburden failure, and the conclusion that ^“^50% filling ratio is insufficient” is overly absolute. It is recommended to add 2–3 intermediate filling ratio tests (if physical tests are not feasible, numerical simulation tests can be used to verify the results), establish quantitative relationship curves among filling ratio, surface subsidence, and key stratum fracture, and determine the critical filling ratio threshold.

**Response:** Thank you for this constructive suggestion. We acknowledge the limitation of only adopting 100 % and 50 % filling‑ratio groups in the current manuscript. We have revised the over‑absolute statements in the Abstract and Conclusions by adding geological‑background restrictions to avoid over‑generalized conclusions. As mentioned in the limitation paragraph of Section 4 Discussion, multi‑gradient numerical simulations covering 90 %, 70 % and 60 % will be implemented in follow‑up research to build quantitative curves and identify the critical filling‑ratio threshold under diverse geological conditions.

6. In the PFC numerical simulation tests, the paper lacks an explanation of how the 50% filling ratio is realized and simulated. Whether it is achieved by reducing the number of particles or altering particle strength is not clearly stated, which may directly affect the interpretability of the results.

**Response:** Thank you for this critical comment regarding the realisation method for the 50 % filling‑ratio condition in PFC simulation. We have supplemented explicit descriptive text in Section 3.1.3 Determination of mining method, clarifying that different filling ratios are realised by adjusting the particle‑packing porosity inside the designated filling zones, rather than modifying particle meso‑mechanical parameters or reducing particle size. The gangue particle set (particle‑generation algorithm, particle‑size distribution, contact parameters, particle strength) remains completely identical for both 100 % and 50 % filling‑ratio groups. Only the target void fraction inside filling regions is adjusted to control the filling density, which physically reproduces incomplete on‑site filling with gangue loss. This setup eliminates confounding variables originating from particle property changes and guarantees result comparability.

7. The paper states that “R6 medium-grained sandstone is the main key stratum controlling the movement of the entire overlying strata and load transfer,” but does not explain the identification method and calculation process of the key stratum. It is recommended to supplement specific calculation steps and identification criteria.

**Response:** Thank you for this valuable comment. We have supplemented the key‑stratum identification calculation process and discrimination criteria in Section 2.1 Engineering background, right after the original sentence which identifies R6 medium‑grained sandstone as the main key stratum. We adopt the classical key‑stratum theory: the inter‑layer load calculation formula and the key‑stratum breaking‑sequence discrimination criterion are supplemented; partial intermediate calculation results are given to demonstrate why R6 medium‑grained sandstone is judged as the main key stratum.

8. There is a figure numbering error in Chapter 4. The figure is cited as Fig. 9 in the text, but the figure caption is labeled “Fig. 17”. It should be uniformly corrected to Fig. 9.

**Response:** Thank you for detecting this figure‑numbering mismatch error in Chapter 4. We have corrected the figure caption label from Fig. 17 to Fig. 9. Meanwhile, we checked all cross‑references within Chapter 4, confirming that in‑text citations of this schematic diagram already use Fig. 9. The rest figures in the manuscript are re‑checked sequentially to eliminate further numbering‑disorder risks.

9. The discussion section in Chapter 4 is too brief. It is recommended to add content including comparative discussion with existing research results and analysis of the limitations of the current study.

**Response:** Thank you for this valuable suggestion. We have supplemented two paragraphs at the end of Section 4 Discussion. The first added paragraph carries out comparative discussion between our main findings and published relevant existing research results, clarifying the similarities and incremental differences. The second supplementary paragraph analyses the limitations of the present study, including physical‑model scale effect, simplification in PFC numerical simulation, geological‑condition restriction for the critical filling‑ratio threshold, and corresponding future research directions.

10. The entire paper lacks analysis of the theoretical part. It is recommended to supplement some theoretical analysis content in the main text of the article.

**Response:** Thank you for this important comment. We recognize that the original manuscript mainly relied on physical simulation and PFC numerical simulation results, and the theoretical derivation‑based analysis was relatively weak. We have supplemented targeted theoretical analysis content in Section 4 Discussion. Combined with key‑stratum theory, voussoir beam theory and stress‑arch theory, we carry out theoretical deduction for load transfer mechanism of gangue pillars and the domino‑style chain‑instability triggering condition. The supplementary theoretical analysis connects the observed simulation phenomena with theoretical formula derivation, explains the internal mechanical mechanism of composite three‑arch structure evolution.
