# Supplementary material for: Study on the fracture characteristics controlled by filling ratio in cut-and-fill mining
Source: PLoS One. 2026 Oct 16;21(10):e0354444. doi: 10.1371/journal.pone.0354444 (PMC13674729; doi:10.1371/journal.pone.0354444)
Supplement: Peer review history

# Peer review: Decision Letter 0

**DOI:** 10.1371/journal.pone.0354444.r001

**Authors:** Shengchao Yu

20 Aug 2026

Dear Dr. Wang,

Thank you for submitting your manuscript to PLOS One. After careful consideration, we feel that it has merit but does not fully meet PLOS One’s publication criteria as it currently stands. Therefore, we invite you to submit a revised version of the manuscript that addresses the points raised during the review process.

Please submit your revised manuscript by Oct 19 2026 11:59PM. If you will need more time than this to complete your revisions, please reply to this message or contact the journal office at plosone@plos.org. When you're ready to submit your revision, log on to https://www.editorialmanager.com/pone/ and select the 'Submissions Needing Revision' folder to locate your manuscript file.

- A letter that responds to each point raised by the academic editor and reviewer(s). You should upload this letter as a separate file labeled 'Response to Reviewers'.
- A marked-up copy of your manuscript that highlights changes made to the original version. You should upload this as a separate file labeled 'Revised Manuscript with Track Changes'.
- An unmarked version of your revised paper without tracked changes. You should upload this as a separate file labeled 'Manuscript'.

If you would like to make changes to your financial disclosure, please include your updated statement in your cover letter. Guidelines for resubmitting your figure files are available below the reviewer comments at the end of this letter.

If applicable, we recommend that you deposit your laboratory protocols in protocols.io to enhance the reproducibility of your results. Protocols.io assigns your protocol its own identifier (DOI) so that it can be cited independently in the future. For instructions see: https://journals.plos.org/plosone/s/submission-guidelines#loc-laboratory-protocols. Additionally, PLOS One offers an option for publishing peer-reviewed Lab Protocol articles, which describe protocols hosted on protocols.io. Read more information on sharing protocols at https://plos.org/protocols?utm_medium=editorial-email&utm_source=authorletters&utm_campaign=protocols.

As the corresponding author, your ORCID iD is verified in the submission system and will appear in the published article. PLOS supports the use of ORCID, and we encourage all coauthors to register for an ORCID iD and use it as well. Please encourage your coauthors to verify their ORCID iD within the submission system before final acceptance, as unverified ORCID iDs will not appear in the published article. *Only*  the individual author can complete the verification step; PLOS staff *cannot*  verify ORCID iDs on behalf of authors.

We look forward to receiving your revised manuscript.

Kind regards,

Shengchao Yu, Ph.D.

Academic Editor

PLOS One

Journal Requirements:

When submitting your revision, we need you to address these additional requirements.

1.Please ensure that your manuscript meets PLOS ONE's style requirements, including those for file naming. The PLOS ONE style templates can be found at

https://journals.plos.org/plosone/s/file?id=wjVg/PLOSOne_formatting_sample_main_body.pdf and

https://journals.plos.org/plosone/s/file?id=ba62/PLOSOne_formatting_sample_title_authors_affiliations.pdf

2. Please note that PLOS One has specific guidelines on code sharing for submissions in which author-generated code underpins the findings in the manuscript. In these cases, we expect all author-generated code to be made available without restrictions upon publication of the work. Please review our guidelines at https://journals.plos.org/plosone/s/materials-and-software-sharing#loc-sharing-code and ensure that your code is shared in a way that follows best practice and facilitates reproducibility and reuse.

3. In the online submission form, you indicated that “The datasets generated and analyzed during this study are available from the corresponding author upon reasonable request.”

All PLOS journals now require all data underlying the findings described in their manuscript to be freely available to other researchers, either 1. In a public repository, 2. Within the manuscript itself, or 3. Uploaded as supplementary information.

This policy applies to all data except where public deposition would breach compliance with the protocol approved by your research ethics board. If your data cannot be made publicly available for ethical or legal reasons (e.g., public availability would compromise patient privacy), please explain your reasons on resubmission and your exemption request will be escalated for approval.

4. In the online submission form, you indicated that your data is available only on request from a third party. Please note that your Data Availability Statement is currently missing the name of the third party contact or institution / contact details for the third party, such as an email address or a link to where data requests can be made. Please update your statement with the missing information.

5. Thank you for stating the following in the Competing Interests section:

“The authors have declared that no competing interests exist.”

We note that one or more of the authors are employed by a commercial company: “Qianyingzi Coal Mine, Anhui Hengyuan Coal Industry and Electricity Power Co., Ltd., Wanbei Coal-Electricity Group, Jinfeng Coal Mine, National Energy Group Ningxia Coal Industry Co., Ltd.”

1. Please provide an amended Funding Statement declaring this commercial affiliation, as well as a statement regarding the Role of Funders in your study. If the funding organization did not play a role in the study design, data collection and analysis, decision to publish, or preparation of the manuscript and only provided financial support in the form of authors' salaries and/or research materials, please review your statements relating to the author contributions, and ensure you have specifically and accurately indicated the role(s) that these authors had in your study. You can update author roles in the Author Contributions section of the online submission form.

Please also include the following statement within your amended Funding Statement.

“The funder provided support in the form of salaries for authors “QQ and JW’, but did not have any additional role in the study design, data collection and analysis, decision to publish, or preparation of the manuscript. The specific roles of these authors are articulated in the ‘author contributions’ section.”

If your commercial affiliation did play a role in your study, please state and explain this role within your updated Funding Statement.

2. Please also provide an updated Competing Interests Statement declaring this commercial affiliation along with any other relevant declarations relating to employment, consultancy, patents, products in development, or marketed products, etc.

Within your Competing Interests Statement, please confirm that this commercial affiliation does not alter your adherence to all PLOS ONE policies on sharing data and materials by including the following statement: "This does not alter our adherence to  PLOS ONE policies on sharing data and materials.” (as detailed online in our guide for authors http://journals.plos.org/plosone/s/competing-interests) . If this adherence statement is not accurate and  there are restrictions on sharing of data and/or materials, please state these. Please note that we cannot proceed with consideration of your article until this information has been declared.

Please include both an updated Funding Statement and Competing Interests Statement in your cover letter. We will change the online submission form on your behalf.

6. Please ensure that you refer to Figure 9 in your text as, if accepted, production will need this reference to link the reader to the figure.

7. Please upload a copy of Figure 17, to which you refer in your text on page 22. If the figure is no longer to be included as part of the submission please remove all reference to it within the text.

8. We note that Figure 1 in your submission contain map images which may be copyrighted. All PLOS content is published under the Creative Commons Attribution License (CC BY 4.0), which means that the manuscript, images, and Supporting Information files will be freely available online, and any third party is permitted to access, download, copy, distribute, and use these materials in any way, even commercially, with proper attribution. For these reasons, we cannot publish previously copyrighted maps or satellite images created using proprietary data, such as Google software (Google Maps, Street View, and Earth). For more information, see our copyright guidelines: http://journals.plos.org/plosone/s/licenses-and-copyright.

We require you to either (1) present written permission from the copyright holder to publish these figures specifically under the CC BY 4.0 license, or (2) remove the figures from your submission:

1. You may seek permission from the original copyright holder of Figure 1 to publish the content specifically under the CC BY 4.0 license.

We recommend that you contact the original copyright holder with the Content Permission Form (http://journals.plos.org/plosone/s/file?id=7c09/content-permission-form.pdf) and the following text:

“I request permission for the open-access journal PLOS ONE to publish XXX under the Creative Commons Attribution License (CCAL) CC BY 4.0 (http://creativecommons.org/licenses/by/4.0/). Please be aware that this license allows unrestricted use and distribution, even commercially, by third parties. Please reply and provide explicit written permission to publish XXX under a CC BY license and complete the attached form.”

Please upload the completed Content Permission Form or other proof of granted permissions as an "Other" file with your submission.

In the figure caption of the copyrighted figure, please include the following text: “Reprinted from [ref] under a CC BY license, with permission from [name of publisher], original copyright [original copyright year].”

2. If you are unable to obtain permission from the original copyright holder to publish these figures under the CC BY 4.0 license or if the copyright holder’s requirements are incompatible with the CC BY 4.0 license, please either i) remove the figure or ii) supply a replacement figure that complies with the CC BY 4.0 license. Please check copyright information on all replacement figures and update the figure caption with source information. If applicable, please specify in the figure caption text when a figure is similar but not identical to the original image and is therefore for illustrative purposes only.

The following resources for replacing copyrighted map figures may be helpful:

USGS National Map Viewer (public domain): http://viewer.nationalmap.gov/viewer/

The Gateway to Astronaut Photography of Earth (public domain): http://eol.jsc.nasa.gov/sseop/clickmap/

Maps at the CIA (public domain): https://www.cia.gov/library/publications/the-world-factbook/index.html and https://www.cia.gov/library/publications/cia-maps-publications/index.html

NASA Earth Observatory (public domain): http://earthobservatory.nasa.gov/

Landsat: http://landsat.visibleearth.nasa.gov/

USGS EROS (Earth Resources Observatory and Science (EROS) Center) (public domain): http://eros.usgs.gov/#

Natural Earth (public domain): http://www.naturalearthdata.com/

9. Please update your submission to use the PLOS LaTeX template. The template and more information on our requirements for LaTeX submissions can be found at http://journals.plos.org/plosone/s/latex.

10. If the reviewer comments include a recommendation to cite specific previously published works, please review and evaluate these publications to determine whether they are relevant and should be cited. There is no requirement to cite these works unless the editor has indicated otherwise.

Additional Editor Comments:

The reviewers have positively evaluated the practical and technical value of this manuscript, but substantial revisions are required. The authors must rigorously address the concerns regarding the experimental design (filling ratio gradients and PFC implementation), correct inconsistencies in table data and figure numbering, supplement theoretical calculations for key strata, and improve overall formatting and language quality. The manuscript is recommended for Major Revision.

[Note: HTML markup is below. Please do not edit.]

Reviewer's Responses to Questions

**Comments to the Author**

1. Is the manuscript technically sound, and do the data support the conclusions?

Reviewer #1: Yes

Reviewer #2: Yes

Reviewer #3: Yes

**********

2. Has the statistical analysis been performed appropriately and rigorously?

Reviewer #1: Yes

Reviewer #2: Yes

Reviewer #3: Yes

**********

3. Have the authors made all data underlying the findings in their manuscript fully available?

The PLOS Data policy

Reviewer #1: Yes

Reviewer #2: Yes

Reviewer #3: No

**********

4. Is the manuscript presented in an intelligible fashion and written in standard English?

Reviewer #1: Yes

Reviewer #2: Yes

Reviewer #3: Yes

**********

Reviewer #1: This manuscript systematically studies the overlying strata fracture characteristics and stope instability mechanism under different filling ratios in Cut-and-fill mining by integrating similar material simulation, PFC numerical modeling and theoretical analysis. The research topic is closely aligned with the demand for green coal mining and surface subsidence control, with a clear engineering background and practical application value. However, the manuscript meets the basic publication requirements of the journal and is recommended for publication after minor revision.

1)The abstract covers rich content but is slightly stacked with detailed descriptions. It is suggested to appropriately streamline secondary process descriptions, further highlight core conclusions and key regulatory mechanisms, and improve the conciseness and readability of the abstract.

2)The literature review in the introduction is relatively comprehensive. It is suggested to add a separate paragraph at the end of the introduction to clearly condense the specific innovations of this work and the research gaps it fills, so that readers can quickly grasp the incremental contribution of the paper.

3) Please check and unify the coordinate axis labels and units of Fig.4 to ensure the consistency of marking specifications across the figure; Please check and correct the coordinate axis information of Fig.5 to ensure the accuracy of physical quantity names and unit marks; Appropriately improve the resolution of some figures and optimize the clarity of local labels to meet the standard requirements of journal publication.

4)Please check the consistency of core data and conclusion expression between the abstract and the conclusion section, unify the description of key conclusions, and avoid expression differences, so as to enhance the logical rigor of the full text.

5)It is suggested to carry out a full-text polish on the English writing, correct a small number of unidiomatic collocations and minor grammatical problems, and further improve the preciseness and academic standard of the language.

6)Please uniformly sort out the reference citation format in the text and the list format at the end of the paper.

Reviewer #2: This paper takes cut-and-fill mining as the research object, combines similar material physical simulation and PFC particle flow numerical simulation to reveal the control law of filling ratio on overlying rock fracture and gangue pillar chain instability, and constructs a composite stress arch theoretical model of stope surrounding rock. The research background is clear, the technical route is complete, and the experimental data and theoretical derivation are sufficient. The research results have direct guiding value for on-site filling parameter optimization and surface subsidence control. However, partial text supplementation, parameter explanation and format standardization are needed before accepted.

1. Briefly supplement the core innovation difference between this paper and existing research. Existing studies mostly focus on vertical compaction filling rate, while this paper systematically reveals the spatial-temporal fracture evolution of overburden and domino instability mechanism of gangue pillars under different horizontal filling ratios, add 1–2 short sentences to highlight this novelty.

2. When citing literatures about filling rate and strata control, briefly explain the theoretical basis of using composite stress arch theory to analyze gangue pillar load transfer in this paper, lay a clear foreshadowing for the subsequent theoretical model part.

3. The basis for setting two working conditions (100% and 50% filling ratio) needs a brief supplementary explanation. Please add a small paragraph to illustrate that 100% filling represents the ideal full support working condition in engineering, while 50% filling corresponds to the common incomplete filling, gangue loss scene on site, and clarify the engineering comparative significance of the two groups of tests.

4. Briefly supplement the consideration of selecting 2–5 mm sand as filling aggregate in the physical model, and explain the similarity matching relationship between this particle size and on-site gangue gradation.

5. Supplement a brief description of the particle filling and modeling operation steps in PFC, and briefly explain how the unified particle generation method ensures the comparability of 100% and 50% filling ratio simulation results.

6. Uniformly standardize the captions of all figures (Fig.1–Fig.17), unify capitalization and punctuation rules; check redundant spaces and misaligned labels in SEM/PFC cloud map annotations, trim irregular layout marks.

7. Carefully scan the whole text for typos, redundant spaces and non-standard abbreviations. If professional abbreviations appear for the first time, complete the full name; correct minor spelling errors to improve readability.

8. Briefly supplement the engineering application guidance value of the symmetrical load distribution law of gangue pillars, connect the theoretical calculation conclusion with the on-site layout optimization of middle and end gangue pillars.

Reviewer #3: The article 《Study on the Fracture Characteristics Controlled by Filling Ratio in Cut-and-fill Mining》 focuses on the issues of surface subsidence and stope instability caused by insufficient filling ratio in cut-and-fill mining. Employing an integrated research approach combining similar material simulation, PFC numerical modeling, and theoretical analysis, this study reveals the control mechanism of filling ratio on the deformation and failure of gangue pillars and the spatiotemporal evolution law of overlying strata fracture. By constructing a "composite stress arch" structural model, it elucidates the intrinsic correlation between the evolution of the three-arch structure during gangue compaction and overburden damage as well as surface subsidence. The final results demonstrate that a 100% filling ratio can effectively restrain roof movement, prevent key stratum fracture, and maintain stable loading of gangue pillars, whereas a 50% filling ratio leads to key stratum breakage, prior destabilization of middle gangue pillars, and subsequent chain failure. These findings can provide theoretical basis and engineering reference for filling parameter optimization, dynamic disaster prevention and control, and surface subsidence management in cut-and-fill mining. However, the paper still has the following deficiencies:

1、The references are not cited in accordance with the manuscript formatting requirements. Please reasonably adjust the sequential order of the references.

2、The stratigraphic overview in Table 1 does not correspond to the information in the coal-rock stratum histogram of Fig. 1, and the thicknesses of each coal and rock stratum are inconsistent.

3、The parameters used in the numerical simulation tests in Table 2 do not correspond to those in Table 1. It is recommended to carefully check and revise them.

4、The expression of the unit weight unit in the tables is incorrect. The correct format should be "kN/m³ or kN·m⁻³", rather than "kN.m³".

5、The paper only sets two filling comparison groups (100% and 50%), lacking intermediate gradients such as 70% and 90%. This may fail to properly reveal the quantitative relationship between filling ratio and overburden failure, and the conclusion that "50% filling ratio is insufficient" is overly absolute. It is recommended to add 2–3 intermediate filling ratio tests (if physical tests are not feasible, numerical simulation tests can be used to verify the results), establish quantitative relationship curves among filling ratio, surface subsidence, and key stratum fracture, and determine the critical filling ratio threshold.

6、In the PFC numerical simulation tests, the paper lacks an explanation of how the 50% filling ratio is realized and simulated. Whether it is achieved by reducing the number of particles or altering particle strength is not clearly stated, which may directly affect the interpretability of the results.

7、The paper states that "R6 medium-grained sandstone is the main key stratum controlling the movement of the entire overlying strata and load transfer," but does not explain the identification method and calculation process of the key stratum. It is recommended to supplement specific calculation steps and identification criteria.

8、There is a figure numbering error in Chapter 4. The figure is cited as Fig. 9 in the text, but the figure caption is labeled "Fig. 17". It should be uniformly corrected to Fig. 9.

9、The discussion section in Chapter 4 is too brief. It is recommended to add content including comparative discussion with existing research results and analysis of the limitations of the current study.

10、The entire paper lacks analysis of the theoretical part. It is recommended to supplement some theoretical analysis content in the main text of the article.

**********

what does this mean?). If published, this will include your full peer review and any attached files.

If you choose “no”, your identity will remain anonymous but your review may still be made public.

**Do you want your identity to be public for this peer review?**  For information about this choice, including consent withdrawal, please see our Privacy Policy

Reviewer #1: No

Reviewer #2: No

Reviewer #3: No

**********

[NOTE: If reviewer comments were submitted as an attachment file, they will be attached to this email and accessible via the submission site. Please log into your account, locate the manuscript record, and check for the action link "View Attachments". If this link does not appear, there are no attachment files.]

To ensure your figures meet our technical requirements, please review our figure guidelines: https://journals.plos.org/plosone/s/figures

You may also use PLOS’s free figure tool, NAAS, to help you prepare publication quality figures: https://journals.plos.org/plosone/s/figures#loc-tools-for-figure-preparation.

NAAS will assess whether your figures meet our technical requirements by comparing each figure against our figure specifications.

# Author comment: Author response to Decision Letter 1

**DOI:** 10.1371/journal.pone.0354444.r002

6 Sep 2026

We sincerely appreciate the editor‑in‑charge and the three reviewers for devoting their time and energy to reviewing our manuscript Study on the Fracture Characteristics Controlled by Filling Ratio in Cut‑and‑fill Mining. The insightful and detailed comments are of great value for polishing the academic content, experimental description, figure formatting and language expression of this work. We have responded to every concern item‑by‑item and implemented corresponding modifications in the revised manuscript, with revision marks provided. We would be grateful if you could consider our revised manuscript for publication.

Reviewer 1:

We thank the anonymous reviewer for the thorough review and thoughtful feedback on our manuscript Study on the Fracture Characteristics Controlled by Filling Ratio in Cut‑and‑fill Mining. All comments have been fully considered and addressed in the revised manuscript. The following presents the point‑by‑point responses to the reviewers’ comments.

1. The abstract covers rich content but is slightly stacked with detailed descriptions. It is suggested to appropriately streamline secondary process descriptions, further highlight core conclusions and key regulatory mechanisms, and improve the conciseness and readability of the abstract.

Response: Thank you for this constructive comment. We have revised the abstract accordingly. We removed redundant procedural descriptions of experimental‑simulation phenomena, condensed repetitive micro‑crack evolution narratives, and emphasized the core controlling mechanism of filling ratio, the composite three‑arch structural essence, and practical engineering suggestions. Specific mark‑ups including before‑and‑after comparisons are presented below. The revised abstract enhances logical compactness and highlights major findings without losing key information.

2. The literature review in the introduction is relatively comprehensive. It is suggested to add a separate paragraph at the end of the introduction to clearly condense the specific innovations of this work and the research gaps it fills, so that readers can quickly grasp the incremental contribution of the paper.

Response: Thank you for this valuable suggestion. Following your advice, we have added an independent stand‑alone paragraph at the end of the Introduction section (before the start of Section 2 Similar simulation. This newly‑added paragraph explicitly summarizes the existing research gaps and three core innovations of this study, highlighting the incremental academic contributions of the present work.

3. Please check and unify the coordinate axis labels and units of Fig.4 to ensure the consistency of marking specifications across the figure; Please check and correct the coordinate axis information of Fig.5 to ensure the accuracy of physical quantity names and unit marks; Appropriately improve the resolution of some figures and optimize the clarity of local labels to meet the standard requirements of journal publication.

Response: Thank you for your careful review and valuable suggestions on figure formatting. We have comprehensively revised Figures 4~5 as requested.

4. Please check the consistency of core data and conclusion expression between the abstract and the conclusion section, unify the description of key conclusions, and avoid expression differences, so as to enhance the logical rigor of the full text.

Response: Thank you for this important comment. We have carefully compared the revised abstract and the Conclusions section of the manuscript. We identified several inconsistent descriptive expressions for core findings between abstract and conclusions. We revised the Conclusions section to align terminology, failure‑mechanism description and structural‑model statement with the updated abstract. We unified key statements regarding filling‑ratio effect, gangue‑pillar chain‑instability mechanism and composite three‑arch structure.

5. It is suggested to carry out a full-text polish on the English writing, correct a small number of unidiomatic collocations and minor grammatical problems, and further improve the preciseness and academic standard of the language.

Response: Thank you for your careful comment on English language usage. We have performed careful full‑text polishing for the manuscript. Minor unidiomatic collocations, grammatical flaws, inconsistent terminology and awkward sentence structures have been revised.

6. Please uniformly sort out the reference citation format in the text and the list format at the end of the paper.

Response: Thank you very much for your careful review and valuable suggestion. We have comprehensively sorted out and standardized the in‑text citation format and end‑paper reference list format in the revised manuscript.

Reviewer 2:

We thank the anonymous reviewer for the thorough review and thoughtful feedback on our manuscript Study on the Fracture Characteristics Controlled by Filling Ratio in Cut‑and‑fill Mining. All comments have been fully considered and addressed in the revised manuscript. The following presents the point‑by‑point responses to the reviewers’ comments.

1. Briefly supplement the core innovation difference between this paper and existing research. Existing studies mostly focus on vertical compaction filling rate, while this paper systematically reveals the spatial-temporal fracture evolution of overburden and domino instability mechanism of gangue pillars under different horizontal filling ratios, add 1–2 short sentences to highlight this novelty.

Response: Thank you for your valuable comment. Following your suggestion, we have added two concise sentences in the newly‑added innovation paragraph at the end of Introduction (after original introductory content, before Section 2 Similar simulation). to highlight the core novelty difference from previous studies. We emphasize that most existing investigations focus on vertical compaction‑related filling rate, whereas this work addresses horizontal filling ratio and further reveals overburden spatiotemporal fracture evolution and the domino‑style gangue‑pillar instability mechanism.

2. When citing literatures about filling rate and strata control, briefly explain the theoretical basis of using composite stress arch theory to analyze gangue pillar load transfer in this paper, lay a clear foreshadowing for the subsequent theoretical model part.

Response: Thank you for your constructive comment. We have supplemented a short explanatory paragraph in the literature‑review segment of Section 1 Introduction, right after reviewing existing filling‑rate‑related studies. We briefly interpret the theoretical basis for adopting composite stress‑arch theory to analyse gangue‑pillar load transfer, and clarify why this arch‑based framework is suitable for cut‑and‑fill mining. This revision lays explicit textual foreshadowing for the subsequent composite three‑arch model presented in the Discussion section.

3. The basis for setting two working conditions (100% and 50% filling ratio) needs a brief supplementary explanation. Please add a small paragraph to illustrate that 100% filling represents the ideal full support working condition in engineering, while 50% filling corresponds to the common incomplete filling, gangue loss scene on site, and clarify the engineering comparative significance of the two groups of tests.

Response: Thank you for this valuable suggestion. We have added a short explanatory paragraph in Section 2.2 Experimental design, right after introducing the two parallel‑experiment schemes (100% and 50% filling ratio). The added text clarifies the practical engineering background of the two test conditions: 100 % filling stands for the ideal full‑support condition, whereas 50 % filling simulates frequent on‑site scenarios of incomplete filling and gangue loss. Meanwhile, we state the comparative engineering significance of this two‑group controlled test.

4. Briefly supplement the consideration of selecting 2–5 mm sand as filling aggregate in the physical model, and explain the similarity matching relationship between this particle size and on-site gangue gradation.

Response: Thank you for your constructive comment. We have supplemented a short explanatory sentence segment in Section 2.2 Experimental design, where the filling‑material description is located. We briefly illustrate the rationality for selecting 2‑5 mm sand as filling aggregate, and clarify the geometric‑similarity‑based matching relationship between this model particle size and the gradation of real‑site gangue.

5. Supplement a brief description of the particle filling and modeling operation steps in PFC, and briefly explain how the unified particle generation method ensures the comparability of 100% and 50% filling ratio simulation results.

Response: Thank you for your valuable suggestion. We have supplemented brief descriptions of particle‑filling workflow and modeling operation steps in Section 3.1.3 Determination of mining method. We also explained that the two groups adopt identical particle‑generation algorithm, particle size distribution and initial particle parameters; only the particle‑filling porosity is adjusted to realise 100 % and 50 % filling‑ratio conditions. This unified particle‑generation scheme eliminates particle‑related confounding variables and guarantees the comparability between the two simulation groups.

6. Uniformly standardize the captions of all figures (Fig.1–Fig.17, unify capitalization and punctuation rules; check redundant spaces and misaligned labels in SEM/PFC cloud map annotations, trim irregular layout marks.

Response: Thank you for your careful suggestions on figure formatting. We have comprehensively standardized all figure captions from Fig.1 to Fig.17 following PLOS ONE journal style requirements. We unified capitalization, sentence‑case writing style and terminal punctuation for each figure caption.

7. Carefully scan the whole text for typos, redundant spaces and non-standard abbreviations. If professional abbreviations appear for the first time, complete the full name; correct minor spelling errors to improve readability.

Response: Thank you for this careful proof‑reading suggestion. We have performed a full‑text manual inspection to identify typos, redundant blank spaces, and non‑standard abbreviations. For discipline‑specific abbreviations, the full complete name is supplemented at their first‑time occurrence in the manuscript. Minor spelling errors, run‑on compound‑word mistakes, extra blank characters and inconsistent terminology are corrected.

8. Briefly supplement the engineering application guidance value of the symmetrical load distribution law of gangue pillars, connect the theoretical calculation conclusion with the on-site layout optimization of middle and end gangue pillars.

Response: Thank you for this constructive comment. We have supplemented a short explanatory paragraph at the end of Section 4 Discussion. We elaborate the engineering guiding significance of the symmetrical load‑distribution law of gangue pillars under cut‑and‑fill mining. We further link the theoretical findings regarding load transfer and chain‑instability risk to on‑site layout and reinforcement optimization for middle and end gangue pillars.

Reviewer 3:

We thank the anonymous reviewer for the thorough review and thoughtful feedback on our manuscript Study on the Fracture Characteristics Controlled by Filling Ratio in Cut‑and‑fill Mining. All comments have been fully considered and addressed in the revised manuscript. The following presents the point‑by‑point responses to the reviewers’ comments.

1. The references are not cited in accordance with the manuscript formatting requirements. Please reasonably adjust the sequential order of the references.

Response: Thank you very much for pointing out this problem. We have comprehensively re‑checked the in‑text citation positions and the reference list of the manuscript.

2. The stratigraphic overview in Table 1 does not correspond to the information in the coal-rock stratum histogram of Fig. 1, and the thicknesses of each coal and rock stratum are inconsistent.

Response: Thank you for pointing out the inconsistency between Table 1 and the comprehensive strata histogram in Fig. 1. We have carefully cross‑checked the original borehole geological data. Table 1 retains the original borehole‑measured stratigraphic thickness data as the authoritative source dataset. We have redrawn the comprehensive strata histogram graphic inside Fig. 1, rescaling the vertical thickness of each stratum bar strictly according to each layer’s thickness value given in Table 1. The lithological sequence, stratum serial number and thickness values in Fig. 1 are now fully consistent with Table 1.

3. The parameters used in the numerical simulation tests in Table 2 do not correspond to those in Table 1. It is recommended to carefully check and revise them.

Response: Thank you for pointing out the parameter inconsistency between Table 1 and Table 2. Table 1 presents original borehole‑measured stratigraphic parameters of the engineering site. Table 2 is the simplified rock‑mass parameter set adopted for PFC numerical simulation, where partial lithologies from Table 1 are merged and simplified for calculation efficiency. We have re‑examined the lithology merging logic and mechanical‑parameter values. We have revised Table 2, making each lithological group’s mechanical parameters correspond reasonably to the weighted average of the matching original borehole strata in Table 1.

4. The expression of the unit weight unit in the tables is incorrect. The correct format should be “kN/m3 or kN·m⁻3”, rather than “kN.m3”.

Response: Thank you for pointing out the wrong format of unit‑weight symbols in tables. We have corrected the erroneous unit symbol kN.m3 to the standard format kN/m3 in Table 1 and Table 2.

5. The paper only sets two filling comparison groups (100% and 50%, lacking intermediate gradients such as 70% and 90%. This may fail to properly reveal the quantitative relationship between filling ratio and overburden failure, and the conclusion that “50% filling ratio is insufficient” is overly absolute. It is recommended to add 2–3 intermediate filling ratio tests (if physical tests are not feasible, numerical simulation tests can be used to verify the results), establish quantitative relationship curves among filling ratio, surface subsidence, and key stratum fracture, and determine the critical filling ratio threshold.

Response: Thank you for this constructive suggestion. We acknowledge the limitation of only adopting 100 % and 50 % filling‑ratio groups in the current manuscript. We have revised the over‑absolute statements in the Abstract and Conclusions by adding geological‑background restrictions to avoid over‑generalized conclusions. As mentioned in the limitation paragraph of Section 4 Discussion, multi‑gradient numerical simulations covering 90 %, 70 % and 60 % will be implemented in follow‑up research to build quantitative curves and identify the critical filling‑ratio threshold under diverse geological conditions.

6. In the PFC numerical simulation tests, the paper lacks an explanation of how the 50% filling ratio is realized and simulated. Whether it is achieved by reducing the number of particles or altering particle strength is not clearly stated, which may directly affect the interpretability of the results.

Response: Thank you for this critical comment regarding the realisation method for the 50 % filling‑ratio condition in PFC simulation. We have supplemented explicit descriptive text in Section 3.1.3 Determination of mining method, clarifying that different filling ratios are realised by adjusting the particle‑packing porosity inside the designated filling zones, rather than modifying particle meso‑mechanical parameters or reducing particle size. The gangue particle set (particle‑generation algorithm, particle‑size distribution, contact parameters, particle strength) remains completely identical for both 100 % and 50 % filling‑ratio groups. Only the target void fraction inside filling regions is adjusted to control the filling density, which physically reproduces incomplete on‑site filling with gangue loss. This setup eliminates confounding variables originating from particle property changes and guarantees result comparability.

7. The paper states that “R6 medium-grained sandstone is the main key stratum controlling the movement of the entire overlying strata and load transfer,” but does not explain the identification method and calculation process of the key stratum. It is recommended to supplement specific calculation steps and identification criteria.

Response: Thank you for this valuable comment. We have supplemented the key‑stratum identification calculation process and discrimination criteria in Section 2.1 Engineering background, right after the original sentence which identifies R6 medium‑grained sandstone as the main key stratum. We adopt the classical key‑stratum theory: the inter‑layer load calculation formula and the key‑stratum breaking‑sequence discrimination criterion are supplemented; partial intermediate calculation results are given to demonstrate why R6 medium‑grained sandstone is judged as the main key stratum.

8. There is a figure numbering error in Chapter 4. The figure is cited as Fig. 9 in the text, but the figure caption is labeled “Fig. 17”. It should be uniformly corrected to Fig. 9.

Response: Thank you for detecting this figure‑numbering mismatch error in Chapter 4. We have corrected the figure caption label from Fig. 17 to Fig. 9. Meanwhile, we checked all cross‑references within Chapter 4, confirming that in‑text citations of this schematic diagram already use Fig. 9. The rest figures in the manuscript are re‑checked sequentially to eliminate further numbering‑disorder risks.

9. The discussion section in Chapter 4 is too brief. It is recommended to add content including comparative discussion with existing research results and analysis of the limitations of the current study.

Response: Thank you for this valuable suggestion. We have supplemented two paragraphs at the end of Section 4 Discussion. The first added paragraph carries out comparative discussion between our main findings and published relevant existing research results, clarifying the similarities and incremental differences. The second supplementary paragraph analyses the limitations of the present study, including physical‑model scale effect, simplification in PFC numerical simulation, geological‑condition restriction for the critical filling‑ratio threshold, and corresponding future research directions.

10. The entire paper lacks analysis of the theoretical part. It is recommended to supplement some theoretical analysis content in the main text of the article.

Response: Thank you for this important comment. We recognize that the original manuscript mainly relied on physical simulation and PFC numerical simulation results, and the theoretical derivation‑based analysis was relatively weak. We have supplemented targeted theoretical analysis content in Section 4 Discussion. Combined with key‑stratum theory, voussoir beam theory and stress‑arch theory, we carry out theoretical deduction for load transfer mechanism of gangue pillars and the domino‑style chain‑instability triggering condition. The supplementary theoretical analysis connects the observed simulation phenomena with theoretical formula derivation, explains the internal mechanical mechanism of composite three‑arch structure evolution.

Attachment Submitted filename: Response to Reviewers.docx

# Peer review: Decision Letter 1

**DOI:** 10.1371/journal.pone.0354444.r003

**Authors:** Shengchao Yu

27 Sep 2026

Study on the Fracture Characteristics Controlled by Filling Ratio in Cut-and-fill Mining

PONE-D-26-33943R1

Dear Dr. Wang,

We’re pleased to inform you that your manuscript has been judged scientifically suitable for publication and will be formally accepted for publication once it meets all outstanding technical requirements.

Within one week, you’ll receive an e-mail detailing the required amendments. When these have been addressed, you’ll receive a formal acceptance letter and your manuscript will be scheduled for publication.

An invoice will be generated when your article is formally accepted. Please note, if your institution has a publishing partnership with PLOS and your article meets the relevant criteria, all or part of your publication costs will be covered. Please make sure your user information is up-to-date by logging into Editorial Manager at Editorial Manager® and clicking the ‘Update My Information' link at the top of the page. For questions related to billing, please contact billing support.

If your institution or institutions have a press office, please notify them about your upcoming paper to help maximize its impact. If they’ll be preparing press materials, please inform our press team as soon as possible -- no later than 48 hours after receiving the formal acceptance. Your manuscript will remain under strict press embargo until 2 pm Eastern Time on the date of publication. For more information, please contact onepress@plos.org.

Kind regards,

Shengchao Yu, Ph.D.

Academic Editor

PLOS One

Additional Editor Comments (optional):

Reviewers' comments:

Reviewer's Responses to Questions

**Comments to the Author**

Reviewer #1: (No Response)

Reviewer #3: All comments have been addressed

**********

2. Is the manuscript technically sound, and do the data support the conclusions?

Reviewer #1: (No Response)

Reviewer #3: Yes

**********

3. Has the statistical analysis been performed appropriately and rigorously?

Reviewer #1: (No Response)

Reviewer #3: Yes

**********

4. Have the authors made all data underlying the findings in their manuscript fully available?

The PLOS Data policy

Reviewer #1: (No Response)

Reviewer #3: Yes

**********

5. Is the manuscript presented in an intelligible fashion and written in standard English?

Reviewer #1: (No Response)

Reviewer #3: Yes

**********

Reviewer #1: The authors have revised the manuscript in accordance with the reviewers' comments. I recommend acceptance.

Reviewer #3: The authors have revised the manuscript in accordance with the reviewers' comments. I recommend acceptance.

**********

what does this mean?). If published, this will include your full peer review and any attached files.

If you choose “no”, your identity will remain anonymous but your review may still be made public.

**Do you want your identity to be public for this peer review?**  For information about this choice, including consent withdrawal, please see our Privacy Policy

Reviewer #1: No

Reviewer #3: No

**********

# Editor report: Acceptance letter

**DOI:** 10.1371/journal.pone.0354444.r004

**Authors:** Shengchao Yu

PONE-D-26-33943R1

PLOS One

Dear Dr. Wang,

I'm pleased to inform you that your manuscript has been deemed suitable for publication in PLOS One. Congratulations! Your manuscript is now being handed over to our production team.

At this stage, our production department will prepare your paper for publication. This includes ensuring the following:

* All references, tables, and figures are properly cited

* All relevant supporting information is included in the manuscript submission,

* There are no issues that prevent the paper from being properly typeset

You will receive further instructions from the production team, including instructions on how to review your proof when it is ready. Please keep in mind that we are working through a large volume of accepted articles, so please give us a few days to review your paper and let you know the next and final steps.

Lastly, if your institution or institutions have a press office, please let them know about your upcoming paper now to help maximize its impact. If they'll be preparing press materials, please inform our press team within the next 48 hours. Your manuscript will remain under strict press embargo until 2 pm Eastern Time on the date of publication. For more information, please contact onepress@plos.org.

You will receive an invoice from PLOS for your publication fee after your manuscript has reached the completed accept phase. If you receive an email requesting payment before acceptance or for any other service, this may be a phishing scheme. Learn how to identify phishing emails and protect your accounts at https://explore.plos.org/phishing.

If we can help with anything else, please email us at customercare@plos.org.

Thank you for submitting your work to PLOS One and supporting open access.

Kind regards,

PLOS One Editorial Office Staff

on behalf of

Dr. Shengchao Yu

Academic Editor

PLOS One
